# Deletion of endothelial IGFBP5 protects against ischaemic hindlimb injury by promoting angiogenesis

**DOI:** 10.1002/ctm2.1725

**Published:** 2024-06-17

**Authors:** Fei Song, Yu Hu, Yi‐Xiang Hong, Hu Sun, Yue Han, Yi‐Jie Mao, Wei‐Yin Wu, Gang Li, Yan Wang

**Affiliations:** ^1^ Xiamen Cardiovascular Hospital of Xiamen University, School of Medicine, Xiamen University Xiamen China; ^2^ Xiamen Key Laboratory of Cardiovascular Diseases Xiamen China

**Keywords:** angiogenesis, cell proliferation, IGF1/IGF2, IGF1R, IGFBP5

## Abstract

**Background:**

Angiogenesis is critical for forming new blood vessels from antedating vascular vessels. The endothelium is essential for angiogenesis, vascular remodelling and minimisation of functional deficits following ischaemia. The insulin‐like growth factor (IGF) family is crucial for angiogenesis. Insulin‐like growth factor‐binding protein 5 (IGFBP5), a binding protein of the IGF family, may have places in angiogenesis, but the mechanisms are not yet completely understood. We sought to probe whether IGFBP5 is involved in pathological angiogenesis and uncover the molecular mechanisms behind it.

**Methods and results:**

IGFBP5 expression was elevated in the vascular endothelium of gastrocnemius muscle from critical limb ischaemia patients and hindlimb ischaemic (HLI) mice and hypoxic human umbilical vein endothelial cells (HUVECs). In vivo, loss of endothelial IGFBP5 (IGFBP5^EKO^) facilitated the recovery of blood vessel function and limb necrosis in HLI mice. Moreover, skin damage healing and aortic ring sprouting were faster in IGFBP5^EKO^ mice than in control mice. In vitro, the genetic inhibition of IGFBP5 in HUVECs significantly promoted tube formation, cell proliferation and migration by mediating the phosphorylation of IGF1R, Erk1/2 and Akt. Intriguingly, pharmacological treatment of HUVECs with recombinant human IGFBP5 ensued a contrasting effect on angiogenesis by inhibiting the IGF1 or IGF2 function. Genetic inhibition of IGFBP5 promoted cellular oxygen consumption and extracellular acidification rates via IGF1R‐mediated glycolytic adenosine triphosphate (ATP) metabolism. Mechanistically, IGFBP5 exerted its role via E3 ubiquitin ligase Von Hippel‐Lindau (VHL)‐regulated HIF1α stability. Furthermore, the knockdown of the endothelial IGF1R partially abolished the reformative effect of IGFBP5^EKO^ mice post‐HLI.

**Conclusion:**

Our findings demonstrate that IGFBP5 ablation enhances angiogenesis by promoting ATP metabolism and stabilising HIF1α, implying IGFBP5 is a novel therapeutic target for treating abnormal angiogenesis‐related conditions.

## Graphical abstract

1. IGFBP5 expression is elevated in ischaemic limb or hypoxic endothelial cells.

2. Deletion of IGFBP5 in the endothelium inhibits the deterioration of ischaemia‐induced limb salvage and blood perfusion.

3. IGFBP5 negatively regulates tube formation, cell proliferation and cell migration by blocking IGF signalling via phosphorylation of the IGF1R/Akt/Erk1/2 pathways in HUVECs.

4. IGFBP5 exerts these functions by regulating the HIF1α stability via E3 ubiquitin ligase VHL.

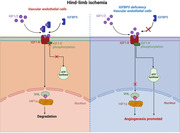

## 1 INTRODUCTION

Vascular injury drives numerous acute and chronic diseases, including critical limb ischaemia (CLI), paediatric stroke, vasospasm and atherosclerosis, which are associated with significant morbidity and mortality.^1^, ^2^, ^3^, ^4^ The condition of CLI is associated with disabled quality of life as well as early cardiovascular and non‐cardiovascular mortality.^5^, ^6^, ^7^, ^8^ Former studies have shown that adeno‐associated viral vector treatment improved survival, motor function and developmental milestones in numerous neural or vascular patients.^9^, ^10^ Thus, it is urgent to develop treatment approaches to eliminate the growth of CLI in patients^11^ and accelerate angiogenesis of ischaemic tissue to restore blood supply.^12^ Hence, a practical therapeutic approach is urgent to establish a remedial way to increase angiogenesis during CLI therapy.

Angiogenesis is essential for physiological conditions and pathological disorders.^13^ Under typical conditions, pro‐ and anti‐angiogenic factors are in balance; when this equilibrium is disrupted, it can lead to abnormal angiogenesis, linked to conditions such as diabetic retinopathy, vascular deformation and cancer.^14^ Hypoxia in poorly perfused tissues is the primary trigger for angiogenesis.^15^ Over the last decades, numerous efforts have been concentrated on developing treatments to revascularise ischaemic tissues or prevent abnormal angiogenesis. Still, clinical trials designed to test the proangiogenic role of vascular endothelial growth factor and fibroblast growth factor have not met expectations.^16^ Although some setbacks may stem from inefficient delivery methods, stimulating the growth of durable and functional vessels is proving more challenging than initially thought. To overcome this, novel strategies, such as transplanting bone marrow‐derived cells or delivering agents that stimulate the formation of distal capillaries and proximal collateral conduit vessels, are crucial to develop.^13^, ^14^, ^16^

Insulin‐like growth factor‐binding protein 5 (IGFBP5) is a secretory protein within the IGFBP family, which comprises IGFBP1 to IGFBP7.^17^ Beyond regulating the insulin‐like growth factor (IGF) activity, IGFBPs perform essential functions independent of IGFs.^18^ As the most conserved member of the IGFBP family, IGFBP5 has additional biological functions that do not calculate IGFs, such as inflammatory response,^19^, ^20^ fibrosis,^21^, ^22^ cell adhesion,^23^ cell migration^24^, ^25^ and cell proliferation.^26^, ^27^ These findings strongly suggested that IGFBP5 is critical for angiogenesis regulation. Nonetheless, the specific mechanism of IGFBP5 in the pathogenesis of ischaemic limbs and the part of IGFBP5 in angiogenesis remains unclear. Our preliminary experimental results showed that IGFBP5 was upregulated in the vascular endothelium of gastrocnemius muscle biopsies from patients with CLI, hindlimb ischaemic (HLI) mice, and hypoxia‐stimulated hypoxic human umbilical vein endothelial cells (HUVECs). We hypothesised that IGFBP5 regulates angiogenesis by regulating endothelial metabolism. To test this hypothesis, we generated endothelial‐specific IGFBP5‐knockout (KO) mice and obtained the first evidence that IGFBP5 regulates angiogenesis via multiple mechanisms, including promoting glycolytic metabolism via IGF1R phosphorylation and increasing HIF1α stability via inhibiting E3 ubiquitin ligase Von Hippel‐Lindau (VHL). These results suggested that IGFBP5 is a possible pharmacological target for treating ischaemia‐related diseases.

## 2 METHODS

### 2.1 Reagents and antibodies

Recombinant human IGFBP‐5 (rhIGFBP5; Cat#: 875‐B5‐025) and human IGF‐II/IGF2 (Cat#: 292‐G2‐050) were obtained from R&D Systems. hIGF‐I (Cat#: 8917SC) was purchased from CST. The haematoxylin‒eosin (H&E) kit (Cat#: G1120) and TUNEL apoptosis assay kit (Cat#: T2190) were purchased from Solarbio. The Picro Solarbio Sirius Red Stain Kit (Cat#: ab245887) was obtained from Abcam. TaliTM Cell Cycle Kit (Cat#: A10798), lipofectamine RNAiMAX (Cat#: 13778150) and lipofectamine 3000 (Cat#: L3000008) were purchased from Thermo Fisher. Cell‐Light 5‐ethynyl‐2′‐deoxyuridine (EdU) Apollo488 In Vitro Kit (Cat#: C10310‐3) was obtained from RiboBio. The Seahorse XF Real‐Time ATP Rate Assay Kit (Cat#: 103592‐100) and Seahorse XF Glycolysis Rate Assay Kit (Cat#: 103344‐100) were purchased from Agilent. MG‐132 (Cat#: HY‐13259), cycloheximide (CHX, Cat#: HY‐12320) and picropodophyllin (PPP, Cat#: HY‐15494) were purchased from MedChemExpress (MCE). Endothelial cell medium (ECM, Cat#: 1001) was obtained from ScienCell. Control siRNA (Cat#: sc‐37007), IGFBP5 siRNA (Cat#: sc‐39591), control shRNA (sh‐NC) plasmid (Cat#: sc‐108060) and IGFBP5 shRNA (sh‐IGFBP5) (h) lentiviral (Cat#: sc‐39591‐V) were obtained from Santa Cruz. HB‐AAV2/9‐TIE‐m‐IGF1R‐ZsGreen and HB‐AAV2/9‐TIE‐ZsGreen controls were ordered from HANBIO. Matrigel matrix (Cat#: 356230) was purchased from Corning Life Sciences. Dihydroethidium (DHE, Cat#: S0063) was purchased from Beyotime. CD31 beads (Cat#: 130‐097‐418) were purchased from Miltenyi Biotechnology. The overexpression vector and adenovirus for Ad‐IGFBP5‐HA and Ad‐Control‐HA were purchased from Wuhan Miaoling Biology.

The antibodies used in the present study, including anti‐IGFBP5 (Cat#: sc‐515116, Santa Cruz) and anti‐CD31 Alexa Fluor 647 (Cat#: sc‐376764, Santa Cruz), were used for detecting the IGFBP5 expression and CD31 antigen in the tissue samples. The following antibodies, including anti‐IGFBP5 (Cat#: 10941S, CST), anti‐phospho‐IGF‐I receptor β (Tyr1146) (Cat#: 3021S, CST), anti‐IGF‐I receptor β (Cat#: 9750S, CST), anti‐phospho‐p44/42 MAPK (Erk1/2) (Cat#: 4370S, CST), anti‐p44/42 MAPK (Erk1/2) (Cat#: 4695S, CST), anti‐phospho‐Akt (Ser473) (Cat#: 4060S, CST), anti‐Akt (Cat#: 9272S, CST), anti‐PKM1 (Cat#: 7067S, CST), anti‐PKM2 (Cat#: 4053S, CST), anti‐HIF1α (Cat#: Ab179483, Abcam), anti‐lactate dehydrogenase (LDH, Cat#: Ab52488, Abcam), anti‐VHL (Cat#: Ab270968, Abcam), anti‐Ubiquitin antibody (Cat#: Ab13495, Abcam), anti‐Ubiquitin (K48) antibody (Cat#: Ab140601, Abcam) and anti‐Ubiquitin (K63) antibody (Cat#: Ab179434, Abcam) were used for immunoblotting or immunostaining. Mouse monoclonal β‐actin (Cat#: AC038, ABclonal) was used for loading control.

### 2.2 Animal husbandry

All the mice were kept in the Animal Center of Xiamen University. The animal use and care were according to the Declaration of Helsinki and approved by the Animal Care Committee of Xiamen University (approval no. XMULAC20190120). Mice were anesthetised with inhalation of isoflurane (3%) for surgeries and were euthanised by intoxication with 100% carbon dioxide.

### 2.3 Igfbp5 conditional knockout (CKO)

The Igfbp5 conditional knockout (CKO) strategy was designed and CKO mice were generated by GemPharmatech Co. Ltd. (strain ID: T013063). Igfbp5 contains two transcripts. Based on the structure of the Igfbp5 gene, exons 1−4 of the Igfbp5‐201 transcript, which had all the coding sequences, are suggested as KO regions. This KO region will lead to the disruption of protein function. CRISPR/Cas9 technology was applied to modify the Igfbp5 gene. sgRNA was synthesised in vitro. Cas9, sgRNA and vector were injected into the fertilised eggs of C57BL/6J mice. Fertilised eggs were then transplanted to obtain F0 mice. A stable F1 generation mouse model was established by mating the F0 mice. KO effect was verified through polymerase chain reaction (PCR) and sequencing. After breeding, Igfbp5^flox/flox^ (Igfbp5^f/f^) mice were obtained. We obtained VE‐cadherin‐Cre (Cdh5‐cre) mice (strain#: 017968) from Professor Ren Xu (Xiamen University). IGFBP5^f/f^ mice mated with Cdh5‐cre mice to produce IGFBP5‐EKO mice (IGFBP5^fl/fl^/Cdh5‐cre, IGFBP5^EKO^). The DNA of the mice was extracted from the mice tail and detected by PCR for IGFBP5 WT, loxP‐flanked and Cdh5‐cre alleles. Genotyping primers for the IGFBP5 allele were: (1) 5′ arm: 5′‐CGGATTTCTGAGCCTGGTCTACAG‐3′ (F) and 5′‐GTTGGTATTAGGCTGAGGTCTTGC‐3′ (R) (WT size: 269 bp, targeting size: 373 bp); (2) 3′ arm: 5′‐GAAGTAGGAGCCAGGGTTCTCCT‐3′ (F) and 5′‐AGGCTCCGGTCCACTCTGTAAG‐3′ (R) (WT size: 263 bp; targeting size: 364 bp), and the Cdh5‐Cre allele primers were 5′‐CCAGGCTGACCAAGCTGAG‐3′ (F) and 5′‐CCTGGCGATCCCTGAACA‐3′ (R) (product size: 345 bp). Littermates of IGFBP5^f/f^ and IGFBP5^EKO^ male mice were randomly assigned to the following experiment.

### 2.4 Human samples

Human sample collections were approved by the Institutional Review Board of Xiamen Cardiovascular Hospital of Xiamen University (approval no. 2017‐10), and consent forms were obtained from all patients. Human tissues from the ischaemic limb and the corresponding normal tissues (around the necrotic tissues) were obtained from five patients with arteriosclerosis obliterans of the lower extremities and one patient with iliac artery occlusion who underwent surgery to remove necrotic tissues at the Xiamen Cardiovascular Hospital from May 2022 to April 2022. After collection, these samples were washed with saline and then embedded in the optimal cutting temperature (OCT) compound (Cat#: 4853, Tissue Tek) and stored in a −80°C refrigerator. Tissue sections were obtained using Leica CM1950 microtome for immunofluorescence staining.

### 2.5 Murine hindlimb ischaemia

Age‐matched male control mice (IGFBP5^f/f^) and endothelial cell (EC)‐specific IGFBP5‐KO (IGFBP5^EKO^) mice aged from 8 to 12 weeks were used in this study. Unilateral HLI was induced, as previously reported.^28^ Briefly, mice were anesthetised by isoflurane vaporisation. The entire femoral artery was exposed and ligated using nylon thread. The femoral artery and peripheral branches were then removed. The skin was stitched using discontinuous absorbable sutures. The mice were then individually housed.

For the IGF1R‐related experiments, HB‐AVV2/9‐Tie‐IGF1R‐m (100 μL/mice, 1.3 × 10^12^ vector genomes/mL, HANBIO) or HB‐AAV2/9‐Tie‐control‐m (100 μL/mice, 1.4 × 10^12^ vector genomes/mL, HANBIO) were injected into IGFBP5^EKO^ mice intramuscularly 21 days before the HLI model induction. Blood perfusion was tested using laser Doppler imaging.

On 28 days post‐operative, all mice were euthanised by intoxication with 100% carbon dioxide, and the gastrocnemius muscle of the ischaemic hindlimb was collected for histological analysis. Eight male mice were used in each experimental group and no dead mouse was observed. The total capillary density in the cross‐sections of the ischaemic hindlimb was quantified by staining the tissue slides with anti‐mouse CD31 and a secondary antibody conjugated to horseradish peroxidase.

### 2.6 Laser Doppler‐based tissue perfusion measurement

Blood flow to hindlimbs was tested using a PeriCam PSI Z laser Doppler system (PERIMED). Animals were pre‐warmed to 37°C, with continuous monitoring of their heart rates. Hindlimb blood flow was detected on specific days (days 0, 7, 14, 21 and 28). The reperfusion rate was defined as the ratio of the blood flow in the ipsilateral ischaemic limb to that in the contralateral limb. Perfusion levels in the ischaemic and non‐operated hindlimbs were quantified using the mean pixel value within the region of interest.

### 2.7 Aortic sprouting assay

After euthanasia, the thoracic aortas (1 mm) of IGFBP5^f/f^ and IGFBP5^EKO^ mice were collected, and the surrounding adipose tissue was carefully removed. The thoracic aorta was placed in Matrigel incubated at 37°C and 5% CO_2_. ECM medium (ScienCell) with 5% bovine serum was supplemented to the wells for 7 days. Images were captured on days 4 and 7.

### 2.8 Immunofluorescence staining

The ischaemic limb gastrocnemius tissues from humans or mice were harvested, embedded in OCT compound and stored in a −80°C refrigerator. Cross‐sections (6 μm) were obtained and fixed with acetone for 10 min, permeated with .3% Triton‐X100 TBST for 15 min and incubated with 1% bovine serum albumin (BSA) and 10% donkey serum at room temperature for 60 min to reduce non‐specific binding. Then, the sections were incubated with CD31 Alexa Fluor 647 (1:100, Santa Cruz) or IGFBP5 (1:100, Santa Cruz) antibodies at 4°C overnight. After rinsing three times for 10 min each, the sections were treated with donkey anti‐mouse conjugated with Alexa Fluor 488 (1:200, Abcam) for 1 h. 4'6‐diamidino‐2‐phenylindole (DAPI) (Sigma‒Aldrich) was applied to stain the nucleus (blue). The sections were captured using a fluorescent microscope (Leica).

### 2.9 Histological staining

Sections of murine gastrocnemius muscle tissues were fixed in 4% paraformaldehyde for 10 min and dyed with H&E staining kit (Cat#: G1120, Solarbio) following the manufacturer's instructions. For picro‐sirius red staining, sections were stained using a staining kit (Cat#: ab245887, Abcam) following the instructions. Sections were pictured with a microscope (Leica).

### 2.10 TUNEL and DHE immunofluorescent staining

To determine the level of apoptosis in the gastrocnemius muscle tissue sections, a one‐step TUNEL Cell Apoptosis Detection Kit (Cat#: T2190, Solarbio) was employed. To determine the reactive oxygen species (ROS) levels of the sections, DHE (Beyotime) was added to stain the sections. DAPI was applied to stain the nucleus (blue). Sections were imaged using a fluorescence microscope.

### 2.11 Cutaneous wound healing

To investigate the wound‐healing effect of endothelial KO IGFBP5 in vivo, we performed cutaneous wound healing in age‐matched male IGFBP5^EKO^ and control mice (IGFBP5^f/f^) (8−12 weeks). Mice were anesthetised with isoflurane. The skin on both sides of the dorsal spine was sterilised. A 10‐mm‐diameter hole was produced in the dorsal skin using a perforator to create a bilateral full‐thickness skin wound without damaging the muscles underneath. The sterility was maintained for all surgical procedures. Buprenorphine was administered subcutaneously before the animals were revived from anesthesia. The wound was digitally photographed using a camera at 0, 4 and 7 days. The healing rate was analysed as the relative value of the healed area to the original area of 0 day.

### 2.12 Murine lung EC isolation

Murine lung endothelial cells (MLECs) were collected to determine the efficiency of the KO in IGFBP5^ECO^ mice. After anesthesia, the lungs were removed from the IGFBP5^fl/fl^ and IGFBP5^EKO^ mice and washed in an ice‐cold dulbecco's modified eagle medium (DMEM). Tissue was minced, digested in collagenase type I and shaken for 1 h. After being filtered (with a 70‐μm filter), centrifuged (1500 rpm, 3 min) and resuspended in buffer, cells were incubated with CD31 beads (Cat#: 130‐097‐418, Miltenyi Biotechnology). The cells were then washed and resuspended with a magnetic separator. The cells were transferred to dishes in an ECM medium.

### 2.13 Cell culture

HUVECs were extracted from the umbilical vein of newborns' umbilical cords following the ethical guidelines of Xiamen University, and informed consent was received from all patients involved. The protocol for the isolation has been described previously.^29^, ^30^ HUVECs were cultured in an ECM medium. Cells were infected by IGFBP5‐shRNA (Cat#: sc‐39591‐V, Santa Cruz) and sh‐NC (Cat#: sc‐37007‐V, Santa Cruz) for 72 h, and then puromycin was supplemented to screen for the efficiently transfected cells. For siRNA transfection, HUVECs were transfected with RNAi MAX and IGFBP5‐siRNA or control siRNA for 48 h. Human IGFBP5 recombinant protein was added to HUVECs at 200 ng/mL concentration for 72 h. To investigate the effect of IGF1 (Cat#:8917SC, Cell Signalling Technology) or IGF2 (Cat#: 292‐G2‐050, R&D systems), HUVECs were treated with IGF1 (100 ng/mL) or IGF2 (100 ng/mL) and then incubated with human IGFBP5 recombinant protein (100 ng/mL) or vehicle in serum‐free ECM.

### 2.14 Transcriptomic profiling

To determine the angiogenic target of IGFBP5‐knockdown (KD) in HUVECs, we conducted mRNA sequence analysis of sh‐NC‐ or sh‐IGFBP5‐infected HUVECs. Total RNA was isolated from both groups using TRIzol (15596018, Life Technology). The RNA integrity was tested using an Agilent Bioanalyser 2100. The mRNA libraries were prepared using an Agilent 2100 Bioanalyser. RNA sequencing was carried out on an Illumina NextSeq 500 Bioanalyser and mapped with Gene Ontology (GO) analysis in the GO database (http://www.geneontology.org/). False dicovery rate(FDR)‐adjusted *p*‐value was corrected by the Benjamini‒Hochberg method with FDR ≤.05 as the threshold.

### 2.15 Matrigel tube formation assay

Cell culture plates were pre‐coated with Matrigel matrix (Corning, Cat#: 356230) at 4°C for 15 min, then incubated at 37°C for 60 min. Following a 12‐h serum starvation, HUVECs (2.0 × 10^4^ cells/well) were combined with 1% fetal bovine serum (FBS) ECM and transferred into pre‐coated 96‐well plates. The tube formation capability of HUVECs was assessed for 2‒6 h using microscopy. Tube length was measured using ImageJ software (NIH) plugged into the functional angiogenesis module.

### 2.16 Cell cycle analysis

Cell cycle distribution in HUVECs was assessed using flow cytometry after staining with propidium iodide (PI, Cat#: P1304MP, Thermo Fisher). After infection with shRNA particles or treatment with recombinant proteins, cells were digested with trypsin, rinsed and fixed in ice‐cold ethanol. They were then washed and stained with the PI solution for 30 min. Cell cycle distribution (G0/G1, S and G2/M) in HUVECs was detected using a flow cytometry system (BD Biosciences), and the percentage of cells in each phase was calculated using MODFIT software.

### 2.17 Wound healing assay

Confluent monolayers of HUVECs with different treatments were seeded into six‐well plates. After growing on the monolayer until confluence, a pipette tip was kept perpendicular to the bottom of the dish, and a straight line was produced in the centre of the cell. The cell debris was rinsed off with phosphate‐buffered saline (PBS). Twenty‐four hours later, cells were imaged using a phase‐contrast microscope. Cell mobility was calculated using ImageJ software.

### 2.18 Transwell migration assay

Transwell assay was performed to validate the migratory ability of HUVEC treatment with IGFBP5‐shRNA, rhIGFBP5, rhIGF1 or rhIGF2 using inserts with an 8.0‐μm pore polycarbonate membrane (Corning). Cells (passage 3−8, 2 × 10^4^) in 200 μL of ECM (supplemented with 1% FBS) were placed in the upper chamber, while FBS‐free ECM was in the lower chamber. The non‐migratory cells in the upper chamber were wiped. Cells migrated to the lower side were fixed with 4% paraformaldehyde and stained with crystal violet for 20 min. The migrated cells were counted in five different fields of each well under the phase‐contrast microscope.

### 2.19 Seahorse XF cellular adenosine triphosphate (ATP) rate test

An Agilent Seahorse XF Real‐Time ATP Rate Assay Kit (103592‐100) was applied to detect real‐time ATP rates (glycolytic ATP and mitochondrial ATP) using an Agilent XFe 24 Seahorse Instrument (Agilent). sh‐NC‐ and sh‐IGFBP5‐transfected HUVECs were seeded in XF24 cell culture microplates (5 × 10^4^ cells/well) and incubated at 37°C overnight. Oligomycin (Oligo) and rotenone/antimycin (Rot/AA) drugs were kept at room temperature before use. The instrument was pre‐heated to 37°C before beginning of the assay. The day before the detection, hydrobooster plate was set up with 1 mL of calibrant solution, which was added to the utility plate and then incubated at 37°C in a non‐CO_2_ incubator. The assay medium was prepared with 10 mM D‐glucose, 2 mM glutamine and 1 mM sodium pyruvate. The Seahorse microplate was removed from the incubator, rinsed twice with seahorse assay media and examined under an inverted microscope to ensure cell adhesion. Each well of the microplate was filled with the assay medium (Cat#: 103575‐100) and incubated at 37°C in a non‐CO_2_ incubator for 1.5 h. Oligo and Rot/AA were added to ports A and B of the Hydrobooster plates. After calibration, the microplate was loaded onto the instrument and the assay was performed. The results were analysed using Agilent Seahorse XFe 24 analyser software.

### 2.20 Seahorse XF cellular glycolysis rate test

An Agilent Seahorse XF Glycolysis Rate Assay Kit (Agilent, Cat#: 103344‐100) was applied to determine the rate of glycolysis using an Agilent Seahorse XFe 24 instrument (Agilent). After infection with sh‐NC or sh‐IGFBP5, cells were placed in XF24 cell culture microplates at 5 × 10^4^ cells/well at 37°C. To investigate the role of the IGF1R, sh‐NC and sh‐IGFBP5, HUVECs were treated with PPP (AXK1717, Cat#: HY‐15494, MedChemExpress) for 24 h. Rot/AA (inhibitors of complexes I and III) and 2‐deoxy‐D‐glucose (2‐DG, rate‐limiting enzymes in glycolysis) were kept at room temperature prior to use. The instrument was pre‐heated to 37°C before beginning of the assay. The Hydrobooster plate was prepared the night before, with 1 mL of calibrant solution, which was added to the utility plate, and then incubated at 37°C in a non‐CO_2_ incubator. The assay medium was prepared using the following ingredients: 10 mM D‐glucose, 2 mM glutamine, 1 mM pyruvate and 5 mM HEPES. The wells were examined under an inverted microscope. Each well was filled with assay media and incubated at 37°C in a non‐CO_2_ incubator for 45 min. Rot/AA and 2‐DG stock solutions were prepared. Rot/AA and 2‐DG were added to ports A and B of the Hydrobooster plate. After calibration, the Seahorse microplate was placed in the instrument for detection. The results were analysed using Agilent Seahorse XFe 24 analyser software.

### 2.21 Quantitative real‐time PCR

Total RNA was isolated from hypoxic or normoxic HUVECs using TRIzol (Cat#: 15596026, Life Technology). mRNA levels were determined using an all‐in‐one RT for qPCR kit (Cat#: R333, Vazyme) and SYBR qPCR Master Mix (Cat#: Q712‐02/03, Vazyme). qPCR was performed using the real‐time PCR system (Bio‐Rad). The sequences of primers are as following: Igfbp1—F: 5′‐GAGCACGGAGATAACTGAGGAGG‐3′, R: 5′‐GAGCCTTCGAGCCATCATAGGT‐3′; Igfbp2—F: 5′‐TGTGAGAAGCGCCGGGAC‐3′, R: 5′‐GCCTCCTTCTGAGTGGTCATC‐3′; Igfbp3—F: 5′‐CATCAAGAAAGGGCATGCTAAA‐3′; R: 5′‐GAGGAGAAGTTCTGGGTATCTG‐3′; Igfbp4—F: 5′‐GGGTGTTCTCTTTGGTGTTA‐3′, R: 5′‐TGTTTTTAGGTGGCTGGATG‐3′; Igfbp5—F: 5′‐GTGCTGTGTACCTGCCCAAT‐3′, R: 5′‐CGTCAACGTACTCCATGCCT‐3′; Igfbp6—F: 5′‐GAACCGCAGAGACCAACAGA‐3′, R: 5′‐AGATGGAAGTCTCCAGGGCT‐3′; Igfbp7—F: 5′‐ATTGTATCCTGAGGCTGAGA‐3′, R: 5′‐AAAGGGCACAGTCTAGTGAA‐3′.

### 2.22 Western blot analysis

After the different treatments, cells were lysed in a radio‐immunoprecipitation assay (RIPA) lysis buffer. A bicinchoninic acid kit (Solarbio) was employed to determine the protein concentration. Protein samples (20 μg) were separated using SDS‒PAGE and transferred to polyvinylidene fluoride membranes (Bio‐Rad). The membranes were blocked with non‐fat milk and blotted with phospho‐IGF‐I Rβ, IGF‐I Rβ, Akt, phospho‐Akt (Ser473), p44/42 MAPK (Erk1/2), phospho‐p44/42 MAPK (Erk1/2), HIF1α, PKM1, PKM2, LDH, VHL and β‐actin antibodies at dilution ratios ranging from 1:500 to 1:2000. After been incubated with secondary antibodies, the membranes were stained with an ECL reagent (Solarbio) and detected using a chemiluminescence system. The intensity was calculated with the ImageJ software (NIH).

### 2.23 Immunoprecipitation assay and ubiquitylation assays

The sh‐NC‐ or shIGFBP5‐transfected HUVECs were cultured in 10 cm dishes. When the cell density reached 90%, cells were lysed using lysis buffer. The lysates were centrifuged at 13 200 *g* to obtain the cell proteins and then incubated with an anti‐HiF‐1α antibody (Cat#: ab179483, Abcam). The protein‒antibody conjugate was treated with Protein A/G agarose beads (Santa Cruz) for 3–5 h. Subsequently, the samples were boiled in a sample buffer and analysed using immunoblotting with anti‐ubiquitin, anti‐ubiquitin (linkage‐specific K48), anti‐ubiquitin (linkage‐specific K63) and anti‐VHL antibodies.

### 2.24 Protein stability assay

To determine the protein stability, the sh‐NC‐ and sh‐IGFBP5‐transfected HUVECs were incubated with CHX (20 μg/mL, MedChemExpress). Then, the cell samples were collected at 0, 2, 4, 6, 8 and 12 h and extracted by RIPA lysis buffer. The level of HIF1α was tested by Western blotting.

### 2.25 Overexpression of IGFBP5 and MG132 treatment

To overexpress IGFBP5 in HUVECs, we constructed a pCMV‐IGFBP5 (human)−3 × HA‐Neo (Miaoling Bioscience & Technology) plasmid, transfected it into HUVECs using lipofectamine 3000 for 48 h and then detected the level of IGFBP5 by Western blotting. After 48 h of transfection, the cells were incubated with MG132 (25 μM, MCE) for 4 h. Cells were washed with PBS and lysed with RIPA buffer. Cellular protein was collected by centrifugation and subjected to the Western blot with anti‐IGFBP5 and anti‐HIF1α antibodies.

### 2.26 Statistical analysis

Analyses were performed on at least five independent experiments for each treatment, for both in vivo and in vitro experiments. GraphPad Prism (version 9.0) was applied to prepare the graphs. An unpaired two‐tailed Student's *t*‐test was applied to detect statistically significant differences between two groups, and one‐ or two‐way analysis of variance with Tukey's multiple comparisons test was applied for multiple group comparisons. Data are shown as the mean ± SEM. *p*‐Value < .05 was considered statistically significant.

## 3 RESULTS

### 3.1 IGFBP5 expression is elevated in ischaemic tissues and hypoxic endothelial cells

**FIGURE 1:**
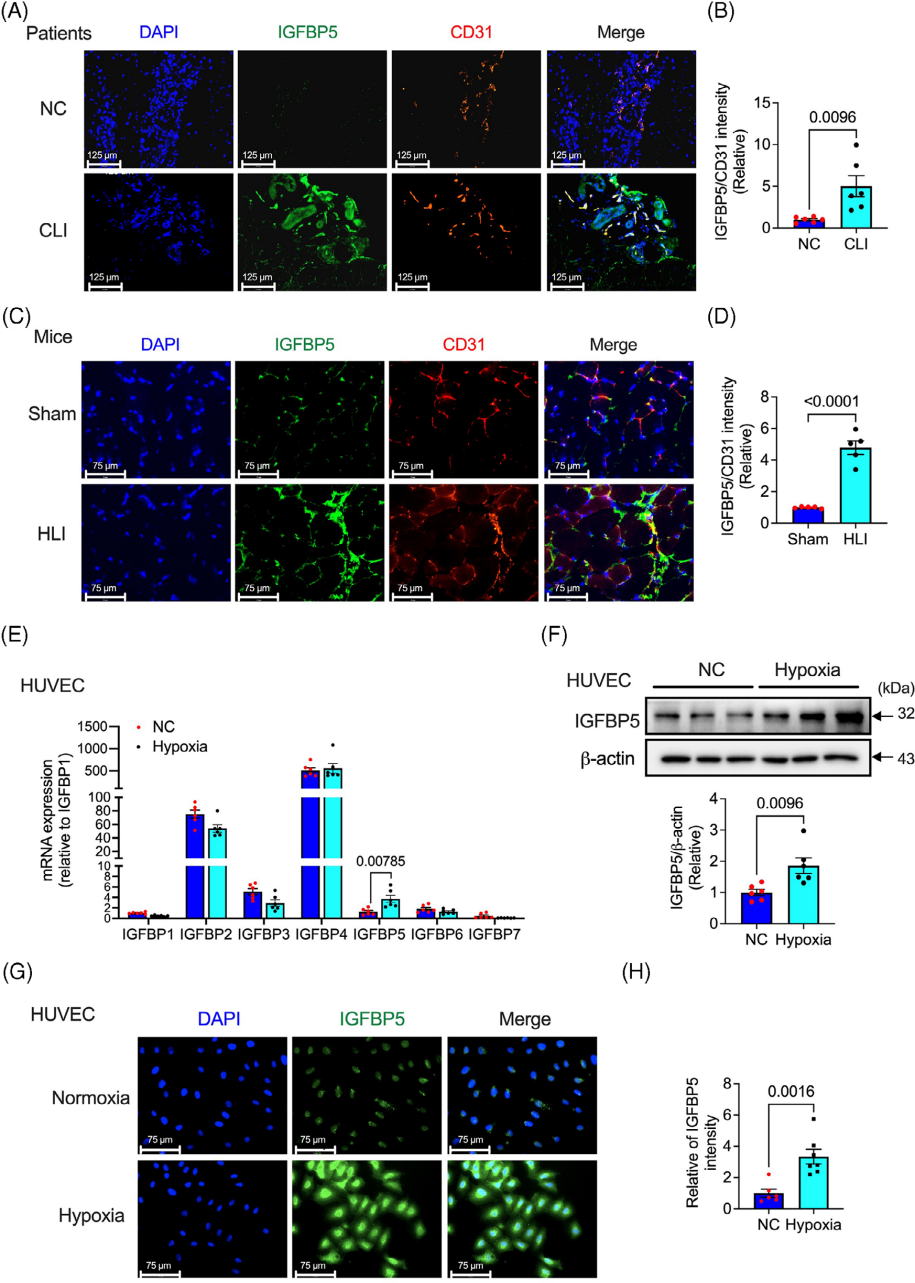
Expression of insulin‐like growth factor‐binding protein 5 (IGFBP5) is elevated in ischaemic tissue or hypoxic human umbilical vein endothelial cells (HUVECs). (A) Representative immunofluorescence images and (B) quantification of the expression of IGFBP5 in ischaemic or non‐ischaemic tissues from patients with critical limb ischaemia (*n* = 6). (C) Representative immunofluorescence images and (D) quantification of the expression of IGFBP5 in gastrocnemius of the ischaemic hindlimb of mice (*n* = 5). (E) mRNA expression of IGFBP1‐7 tested by qRT‐PCR in HUVECs under hypoxia stimulation for 24 h (*n* = 6). (F) Representative Western blotting images and quantification of the expression of IGFBP5 in HUVECs under hypoxia stimulation for 24 h. (G) Representative immunofluorescence images and (H) quantification of the expression of IGFBP5 in HUVECs under hypoxia stimulation for 24 h (*n* = 6).

To explore whether IGFBP5 is critical in angiogenesis, we first determined the level of IGFBP5 in gastrocnemius muscle biopsies from patients with CLI and HLI mice. As shown in Figure 1A,B, the level of IGFBP5 in the gastrocnemius muscle of patients with CLI (detailed information on patients is presented in Table S1) was significantly increased compared to that in non‐ischaemic tissues, which were mainly colocalised with the vascular endothelium. Similar evidence was observed in HLI mice, with increased protein expression levels of IGFBP5 in the ischaemic gastrocnemius muscles around blood vessels compared to sham mice (Figure 1C,D). These results demonstrated that IGFBP5 expression increased after ischaemia and largely overlapped with ischaemia‐induced new capillaries, indicating a possible function of IGFBP5 in ischaemia‐induced neovascularisation. The expression of seven members of IGFBPs was also determined in HUVECs. IGFBP5 was expressed in HUVECs and significantly increased under hypoxia induction (Figure 1E). In contrast, IGFBP2, IGFBP3 and IGFBP4 were highly expressed in HUVECs but did not respond to hypoxia treatment. However, IGFBP1, IGFBP6 and IGFBP7 were barely detectable under basal and hypoxic conditions. Furthermore, IGFBP5 protein was also tested using Western blotting (Figure 1F) and immunofluorescence (Figure 1G,H) in HUVECs under normoxia and hypoxia. We found a significant increase of IGFBP5 expression in response to hypoxia. These results indicate that IGFBP5 is promoted under ischaemic or hypoxic conditions, and its location overlaps with that of ECs, suggesting a crucial role of IGFBP5 in neovascularisation.

### 3.2 EC‐specific depletion of IGFBP5 promotes angiogenesis in HLI mice

To further examine the role of IGFBP5 in ECs, we crossed IGFBP5^flox/flox^ (IGFBP5^f/f^) mice with Cdh5‐Cre mice to produce IGFBP5^flox/flox^/Cdh5‐Cre (IGFBP5^EKO^) mice (Figure S1A). Mouse genotypes were determined by PCR analysis of genomic DNA (Figure S1B). Under normal conditions, IGFBP5^EKO^ mice were viable and fertile and showed no apparent morphological defects or body weight change (Figure S2) compared to control mice (IGFBP5^f/f^). To confirm the effectiveness of IGFBP5‐KO in ECs, we isolated MLECS from control (IGFBP5^f/f^) and EC‐specific IGFBP5‐KO (IGFBP5^EKO^) mice to examine the protein levels of IGFBP5 (Figure S1C). The expression levels of IGFBP5 in MLECs isolated from IGFBP5^EKO^ mice were significantly lower than those in control (IGFBP5^f/f^) (Figure S1).

**FIGURE 2:**
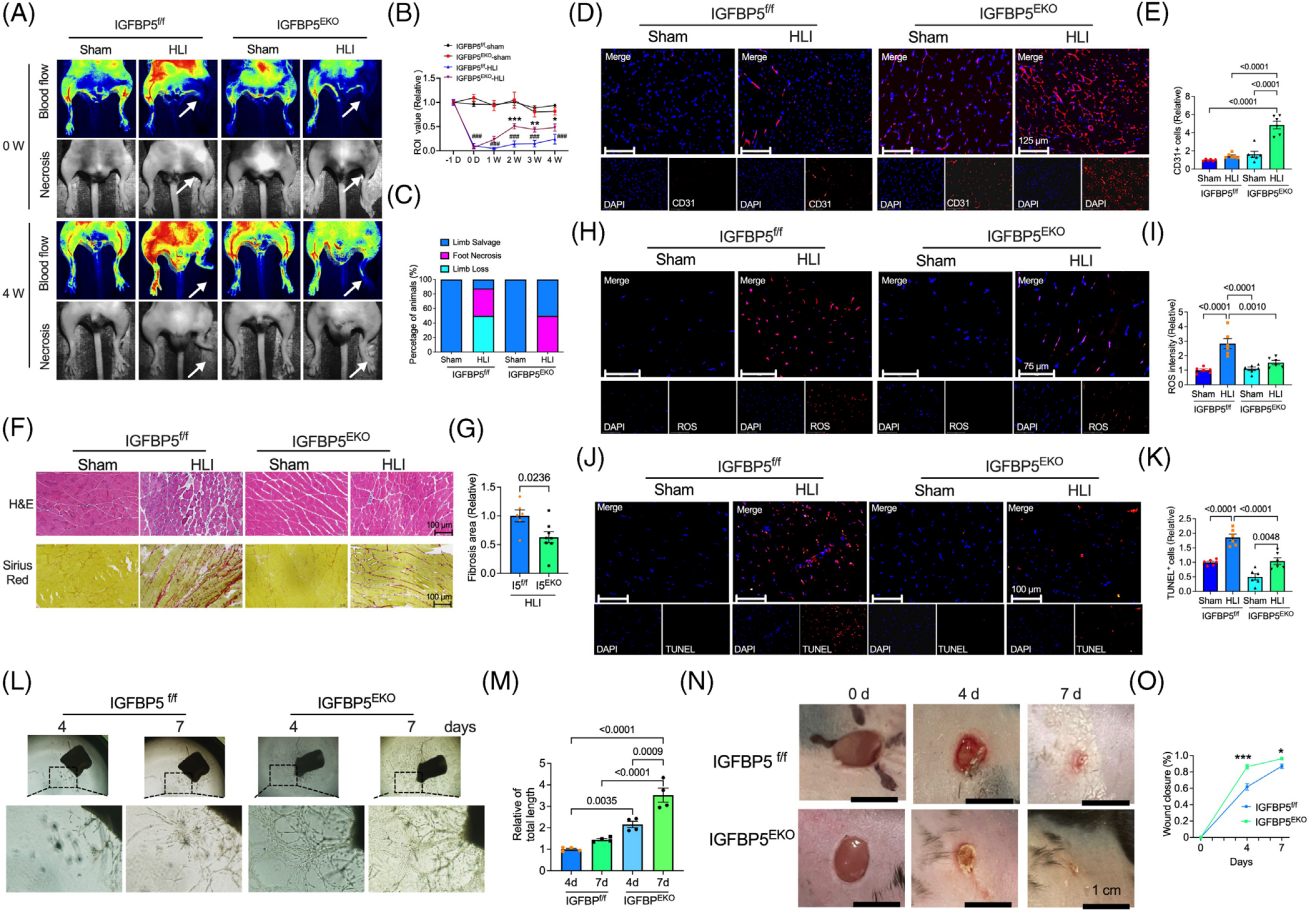
Endothelial cell (EC)‐specific deletion of insulin‐like growth factor‐binding protein 5 (IGFBP5) attenuates the damage of the ischaemic hindlimb in mice. (A) Representative blood flow images of laser Doppler‐based tissue perfusion system in CDH5‐Cre‐IGFBP5^flox/flox^ (IGFBP5^EKO^) and IGFBP5^flox/flox^ (IGFBP5^f/f^) mice after ischaemia induction (0 W) and 4 weeks after ischaemia. (B) Quantification of blood flow in the hindlimb of IGFBP5^EKO^ and IGFBP5^f/f^ mice before (−1 D) and after ischaemia induction at 0, 1, 2, 3 and 4 weeks (*n* = 8 in each group). ^###^ *p* < .001 compared to IGFBP5^f/f^‐sham; ^*^ *p* < .05, ^**^ *p* < .01 and ^***^ *p* < .001 compared to IGFBP5^f/f^‐HLI. (C) Percentage of limb salvage, foot necrosis and limb loss in IGFBP5^EKO^ and IGFBP5^f/f^ mice treated with sham or hindlimb ischaemia (HLI) (*n* = 8 in each group). (D) Representative immunofluorescence staining images and (E) quantification of CD31 (red) in gastrocnemius of hindlimb from IGFBP5^EKO^ and IGFBP5^f/f^ mice treated with sham or HLI (*n* = 5 in each group). (F) Representative haematoxylin‒eosin and picrosirius red staining images and (G) quantification of fibrosis area in gastrocnemius of hindlimb from IGFBP5^EKO^ and IGFBP5^f/f^ mice treated with sham or HLI (*n* = 5 in each group). (H) Representative immunofluorescence staining images and (I) quantification of dihydroethidium (DHE) staining for reactive oxygen species (ROS) in gastrocnemius of hindlimb from IGFBP5^EKO^ and IGFBP5^f/f^ mice treated with sham or HLI (*n* = 5 in each group). (J) Representative immunofluorescence staining images and (K) quantification of TUNEL staining in gastrocnemius of hindlimb from IGFBP5^EKO^ and IGFBP5^f/f^ mice treated with sham or HLI (*n* = 5 in each group). (L) Representative microscopy images of aortic sprouting assay and (M) quantification of tube length of the aorta from IGFBP5^EKO^ and IGFBP5^f/f^ mice at 4 and 7 days after being placed in plates with Matrigel. (N) Representative images and (O) quantification of cutaneous wound healing in IGFBP5^EKO^ and IGFBP5^f/f^ mice at 0, 4 and 7 days after wound induction (^*^ *p* < .05, ^***^ *p* < .001 vs. IGFBP5^f/f^).

We developed the HLI model to substantiate the role of IGFBP5 in neovascularisation. Angiogenesis was assessed by detecting blood flow recovery and the extent of new capillaries in the ischaemic limb. Blood perfusion was monitored before and after the injury. Blood flow in the ischaemic hindlimbs gradually recovered in the control group (IGFBP5^f/f^) and recovered significantly faster in the EC‐IGFBP5‐KO (IGFBP5^EKO^) group than in the controls at 2, 3 and 4 weeks after surgery (Figure 2B). Moreover, we quantified the percentage of mice with limb salvage, foot necrosis and limb loss 4 weeks after surgery. Control (IGFBP5^f/f^) mice exhibited 50% limb loss and 37.5% foot necrosis; however, in IGFBP5^EKO^ mice, limb loss decreased to 0%, and foot necrosis was 50% (Figure 2C).

Neovascularisation and vessel sprouting exhibited a crucial role in restoring blood flow in ischaemic hindlimbs.^31^, ^32^, ^33^, ^34^ Thus, we examined CD31 expression by immunostaining in the ischaemic tissues of IGFBP5^EKO^ and control mice. Immunofluorescence staining of CD31 expression was virtually promoted in the ischaemic gastrocnemius muscles of IGFBP5^EKO^ mice (Figure 2D,E). Interestingly, the IGFBP5‐EKO group showed less inflammation and fibrosis (Figure 2F,G), as well as fewer ROS (Figure 2H,I) and apoptotic myocytes (Figure 2J,K) in the gastrocnemius muscles, compared to the control group. Aortic ring sprouting was used to verify the role of the IGFBP5‐EKO on angiogenesis. As shown in Figure 2L,M, the aortic ring sprouting of IGFBP5^EKO^ mice exhibited significantly increased angiogenesis at 4 and 7 days compared to the control mice (IGFBP5^f/f^), further suggesting the pro‐angiogenic effect of IGFBP5 deletion in vascular ECs. Skin wound healing was also rescued in IGFBP5 ^EKO^ mice compared to control (IGFBP5^f/f^) mice 4 and 7 days after skin wound induction (Figure 2N,O). These findings demonstrated the critical role of IGFBP5‐EC deletion in angiogenesis and repair of ischaemic hindlimbs.

### 3.3 Deficiency of IGFBP5 in HUVECs promotes tube‐like formation, cell proliferation and cell migration

**FIGURE 3:**
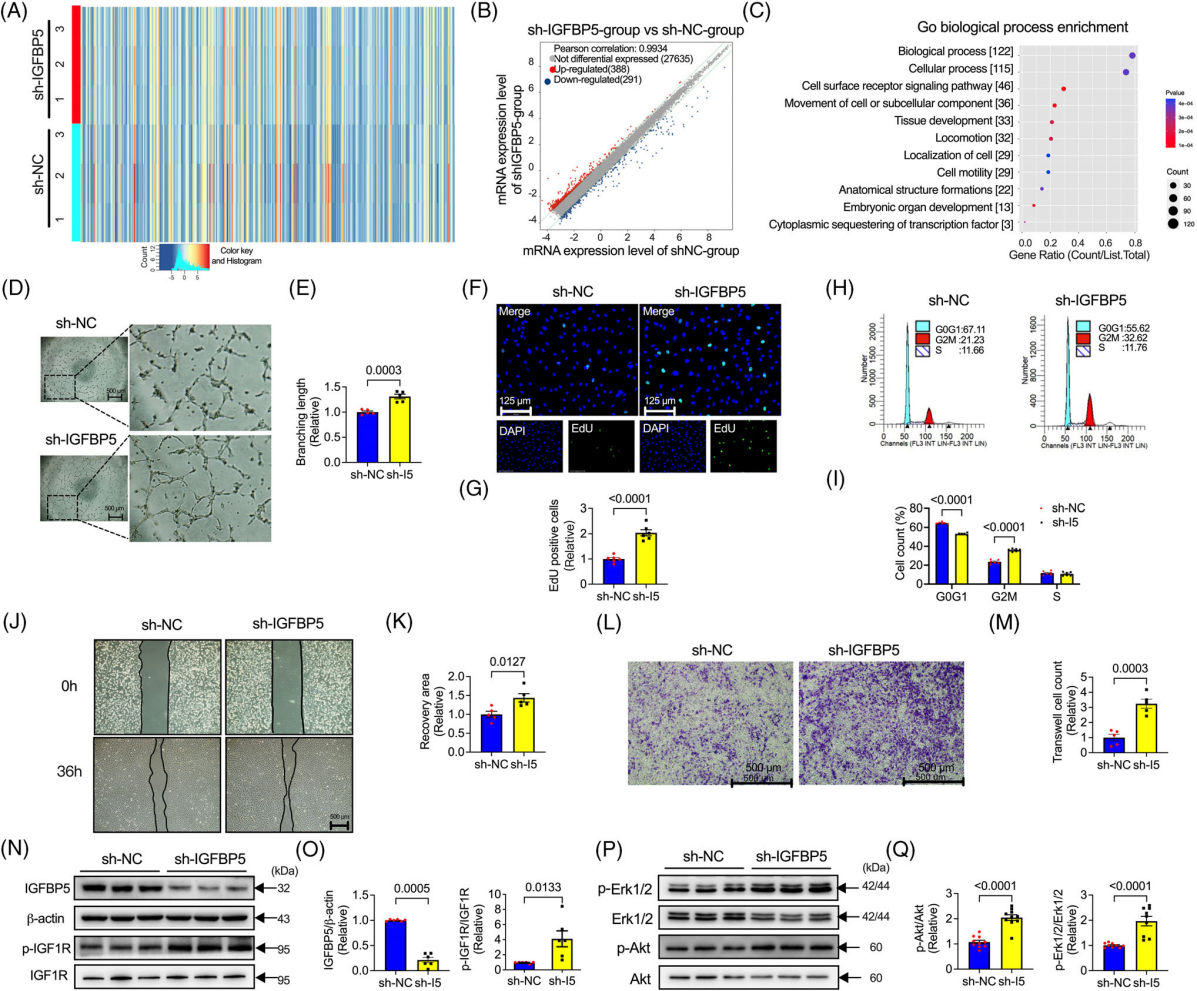
Insulin‐like growth factor‐binding protein 5 (IGFBP5) knockdown promotes tube formation, cell proliferation and migration in human umbilical vein endothelial cells (HUVECs). (A) Representative heatmap, (B) volcano plot and (C) Gene Ontology (GO) biological process enrichment pathway in IGFBP5 shRNA (sh‐IGFBP5)‐ or control shRNA (sh‐NC)‐transfected HUVECs. (D) Representative images and (E) quantification of tube formation in sh‐NC‐ and sh‐IGFBP5‐transfected HUVECs (*n* = 5 in each group). (F) Representative immunofluorescence images and (G) quantification of 5‐ethynyl‐2′‐deoxyuridine (EdU, green)‐stained HUVECs transfected with sh‐NC or sh‐IGFBP5 (*n* = 5 in each group). (H) Representative images and (I) quantification of flow cytometry for cell cycle in sh‐NC‐ or sh‐IGFBP5‐transfected HUVECs (*n* = 5 in each group). (J) Representative images and (K) quantification of wound healing assay in sh‐NC‐ or sh‐IGFBP5‐transfected HUVECs (*n* = 5 in each group). (L) Representative images and (M) quantification of transwell assay in sh‐NC‐ or sh‐IGFBP5‐transfected HUVECs (*n* = 5 in each group). (N) Representative Western blotting images and (O) quantification of IGFBP5, p‐IGF1R and IGF1R expression in sh‐NC‐ or sh‐IGFBP5‐transfected HUVECs. (P) Representative Western blotting images and (Q) quantification of p‐Erk1/2, Erk1/2, p‐Akt and Akt expression in sh‐NC‐ or sh‐IGFBP5‐transfected HUVECs (*n* = 6 in each group).

We investigated the mechanism by which IGFBP5 deficiency leads to angiogenesis. To achieve this, we applied an unbiased RNA sequencing approach to analyse the global landscape of mRNAs in IGFBP5‐KD HUVECs compared to controls. Many differentially expressed genes (DEGs) were observed, and exhibited in the heatmap and volcano plot (Figure 3A,B). Pathway analysis revealed that the DEGs were mainly clustered based on biological processes, cellular processes, cell surface receptor signalling pathways and the movement of cell or subcellular components (Figure 3C). Cellular processes and cell surface receptor signalling pathways play pivotal roles in IGFBP5‐regulated endothelial cell development. A series of assays, including tube formation, cell cycle progression, EdU cell proliferation, transwell assays and wound healing, were applied to examine the role of IGFBP5‐KD on tube formation, cell proliferation and migration in HUVECs. IGFBP5‐KD promoted tube formation (Figure 3D,E) in HUVECs. The cell proliferation examined by EdU assay exhibited a promotion effect in IGFBP5 deficiency HUVECs (Figure 3F,G). Furthermore, cell cycle assay indicated that the KD of IGFBP5 significantly promoted more cells from the G0G1 phase to the G2M phase (Figure 3H,I) than control cells, indicating that IGFBP5‐KD affected the cell cycle progression, which further enhanced the increased proliferation of HUVECs. The wound healing and transwell assays displayed that IGFBP5‐KD increased HUVEC migration (Figure 3J–M).

Mechanistically, since the activation of the IGF1R is critical for regulating the IGF signalling pathway,^35^ we explored IGF1R phosphorylation in IGFBP5‐shRNA HUVECs and found that IGFBP5‐KD significantly promoted the phosphorylation of IGF1R. Previous research by our and other groups has reported that Erk1/2 and Akt phosphorylation are critical for augmenting endothelial or other cell proliferation or migration.^13^, ^14^, ^30^, ^36^, ^37^ Accordingly, we investigated whether these genes were involved in the angiogenic actions of IGFBP5 deletion. We found that the phosphorylation of IGF1R, Erk1/2 and Akt was promoted markedly following IGFBP5‐KD (Figure 3N–Q). These findings suggested that in vitro KD of IGFBP5 regulates IGF1R, Erk1/2 and Akt phosphorylation and is essential for ECs' angiogenic properties, such as cell proliferation and migration.

### 3.4 rhIGFBP5 decreased angiogenic phenotype in HUVECs

**FIGURE 4:**
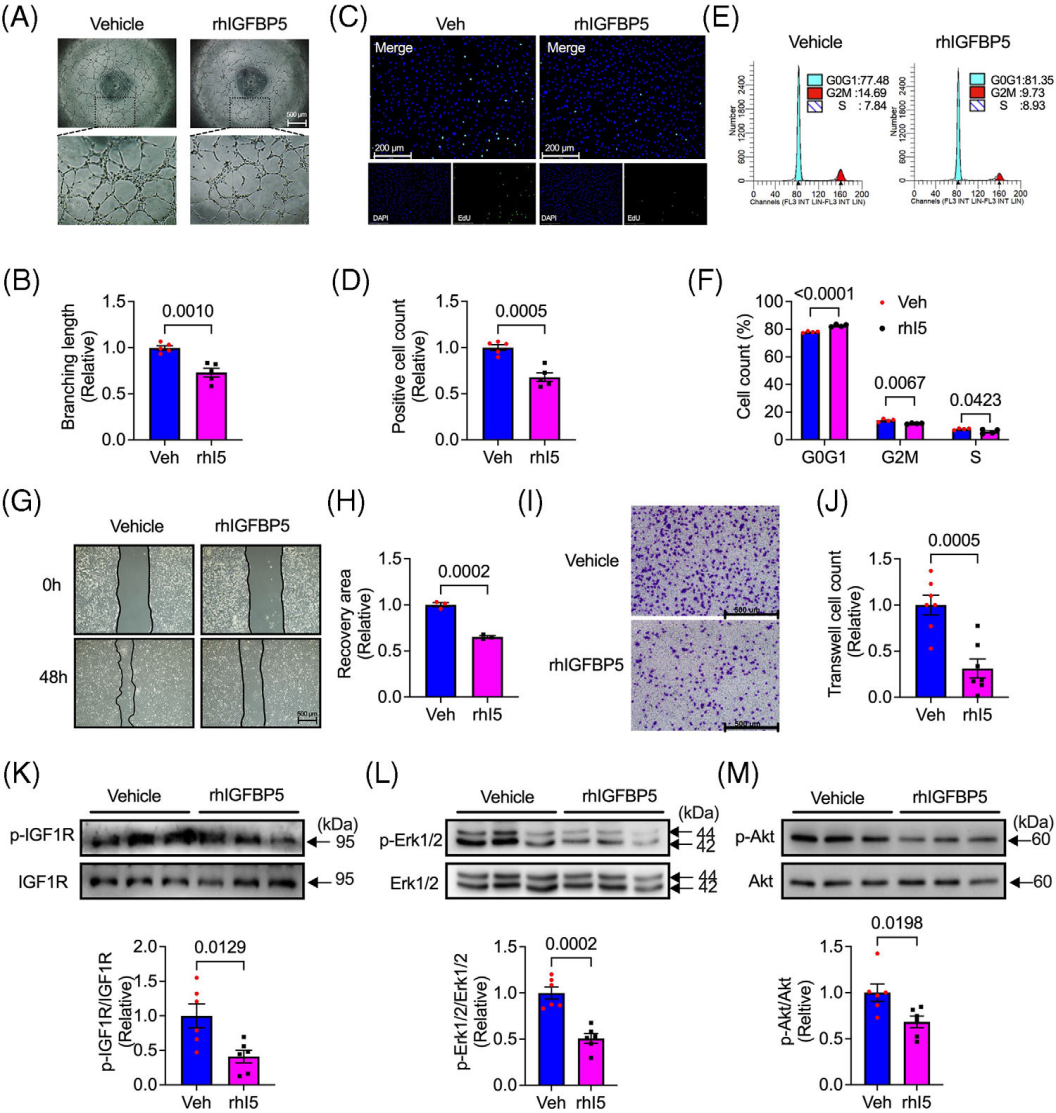
Recombinant human insulin‐like growth factor‐binding protein 5 (IGFBP5) suppresses tube formation, cell proliferation and migration. (A) Representative images and (B) quantification of tube formation in recombinant human IGFBP5 (rhIGFBP5)‐ or vehicle‐treated human umbilical vein endothelial cells (HUVECs). (C) Representative immunofluorescence images and (G) quantification of 5‐ethynyl‐2′‐deoxyuridine (EdU, green)‐stained HUVECs treated with rhIGFBP5 or vehicle (*n* = 5 in each group). (E) Representative images and (F) quantification of flow cytometry for cell cycle in rhIGFBP5‐ or vehicle‐treated HUVECs (*n* = 5 in each group). (G) Representative images and (H) quantification of wound healing assay in rhIGFBP5‐ or vehicle‐treated HUVECs. (I) Representative images and (J) quantification of transwell assay in rhIGFBP5‐ or vehicle‐treated HUVECs (*n* = 5 in each group). Representative Western blotting images and quantification of (K) p‐IGF1R and IGF1R, (L) p‐Erk1/2 and Erk1/2 and (M) p‐Akt, and Akt in rhIGFBP5‐ or vehicle‐treated HUVECs (*n* = 6 in each group).

Furthermore, we explored the role of IGFBP5 overexpression in tube formation, cell proliferation and migration in HUVECs. Because IGFBP5 is a secreted protein, we used rhIGFBP5 for these tests. After treatment for 24 h, the concentration of IGFBP5 still showed a high level (Figure S3A). In contrast to IGFBP5‐KD, pharmacological administration of rhIGFBP5 inhibited tube formation, cell proliferation, cell cycle progression, wound healing and transwell (Figure 4A–J). Intriguingly, the phosphorylation of IGF1R (Figure 4K,L), Erk1/2 (Figure 4M,N) and Akt (Figure 4O,P) was also suppressed by the administration of rhIGFBP5. These results demonstrated the suppression effect of IGFBP5 overexpression on angiogenic genotype in ECs.

### 3.5 rhIGFBP5 suppressed IGF1‐ or IGF2‐promoted tube‐like formation, cell proliferation and migration

**FIGURE 5:**
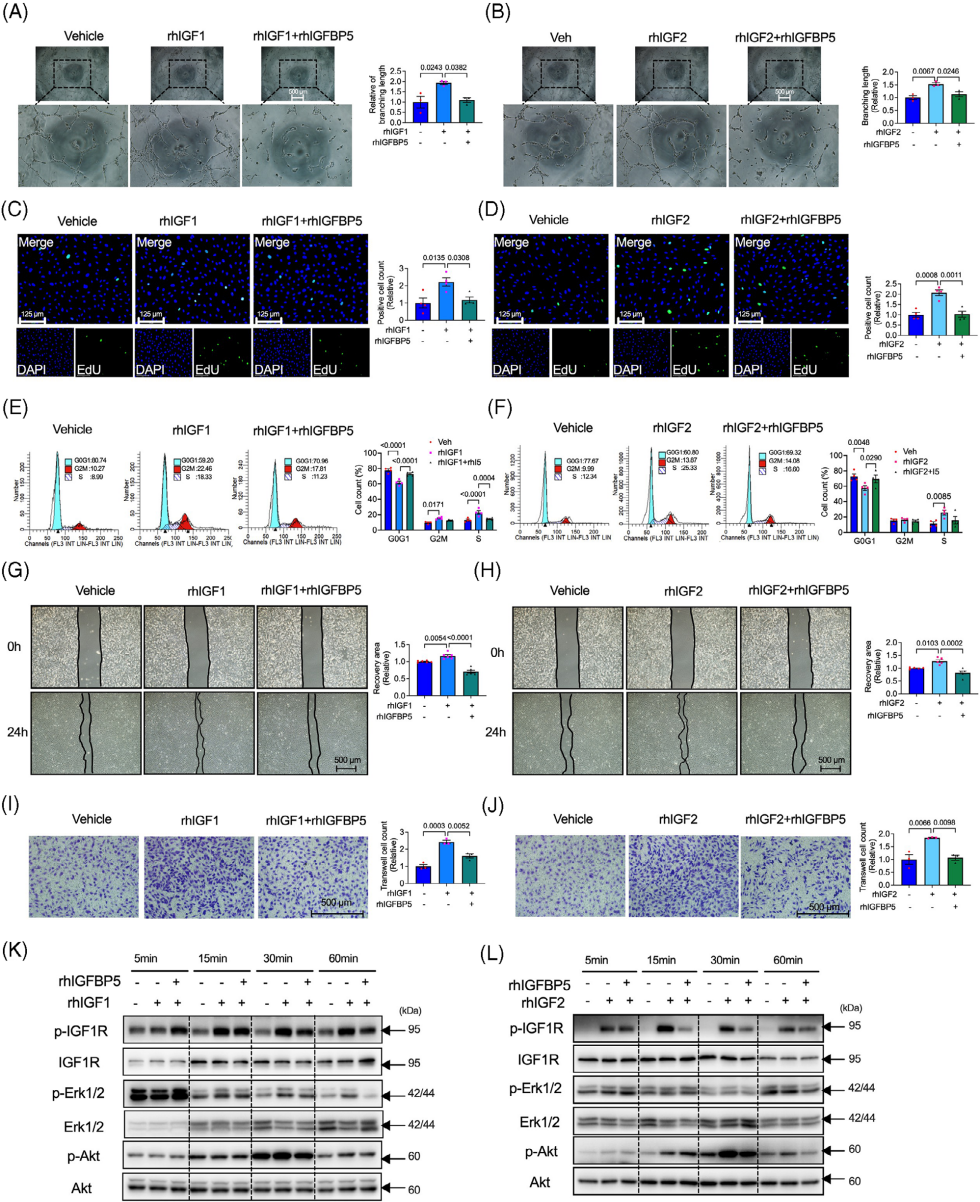
Recombinant human insulin‐like growth factor‐binding protein 5 (IGFBP5) restrains IGF1‐ or IGF2‐induced tube formation, cell proliferation and migration. (A) Representative tube formation images and quantification of human umbilical vein endothelial cells (HUVECs) treated with rhIGF1 in the presence or absence of recombinant human IGFBP5 (rhIGFBP5) (*n* = 5 in each group). (B) Representative tube formation images and quantification of HUVECs treated with rhIGF2 in the presence or absence of rhIGFBP5 (*n* = 5 in each group). (C) Representative immunofluorescence images and quantification of 5‐ethynyl‐2′‐deoxyuridine (EdU, green)‐stained HUVECs treated with rhIGF1 in the presence or absence of rhIGFBP5 (*n* = 5 in each group). (D) Representative immunofluorescence images and quantification of EdU (green)‐stained HUVECs treated with rhIGF2 in the presence or absence of rhIGFBP5 (*n* = 5 in each group). (E) Representative images and quantification of flow cytometry for cell cycle of HUVECs treated with rhIGF1 in the presence or absence of rhIGFBP5 (*n* = 5 in each group). (F) Representative images and quantification of flow cytometry for cell cycle of HUVECs treated with rhIGF2 in the presence or absence of rhGFBP5 (*n* = 5 in each group). (G) Representative images and quantification of wound healing assay of HUVECs treated with rhIGF1 in the presence or absence of rhIGFBP5 (*n* = 5 in each group). (H) Representative images and quantification of wound healing assay of HUVECs treated with rhIGF2 in the presence or absence of rhIGFBP5 (*n* = 5 in each group). (I) Representative images and quantification of transwell assay of HUVECs treated with rhIGF1 in the presence or absence of rhIGFBP5 (*n* = 5 in each group). (J) Representative images and quantification of transwell assay of HUVECs treated with rhIGF2 in the presence or absence of hIGFBP5 (*n* = 5 in each group). (K) Representative images of Western blotting assay‐detected time‐course of p‐IGF1R, IGF1R, p‐Erk1/2, Erk1/2, p‐Ak and Akt expression of HUVECs treated with rhIGF1 in the presence or absence of rhIGFBP5. (L) Representative images of Western blotting assay‐detected time‐course of p‐IGF1R, IGF1R, p‐Erk1/2, Erk1/2, p‐Ak and Akt expression of HUVECs treated with rhIGF2 in the presence or absence of rhIGFBP5.

Although a previous study demonstrates that IGFBP5 reduces the survival‐promoting downstream effects of IGF1 in motoneurons,^38^ the role of IGFBP5 on IGF1 or IGF2 in HUVECs' proliferation and migration has not yet been explored. Our results showed that recombinant human rhIGF1 or rhIGF2 promoted the tube‐like formation (Figure 5A,B), cell proliferation (Figure 5C,D), cell cycle progression (Figure 5E,F) and cell migration (Figure 5G–J) in HUVECs, which were significantly decreased by rhIGFBP5. A molecular pathway dependent on the IGF1, Erk1/2 and Akt phosphorylation (Figure 5K,L) was also observed over time. The phosphorylation of IGF1R was promoted by rhIGF1 at 15 min and rhIGF2 at 5 min; rhIGFBP5 inhibited at 30 min in rhIGF1 treatment and at 15 min in rhIGF2 treatment, respectively. Similar to IGF1R phosphorylation, Erk1/2 phosphorylation was activated 15 min after rhIGF1 treatment and 5 min after rhIGF2 treatment and was suppressed by rhIGFBP5 at 15 and 60 min. Additionally, phosphorylation of Akt was promoted at 30 and 15 min after treatment with rhIGF1 and rhIGF2, respectively, and was suppressed at 30 min by IGFBP5 (Figure 5K,L). These findings indicated that the function of IGFBP5 associated with cell proliferation and migration is regulated by or partially relies on regulating IGFs.

### 3.6 IGFBP5 regulates the glycolytic capacity in HUVECs via IGF1R

**FIGURE 6:**
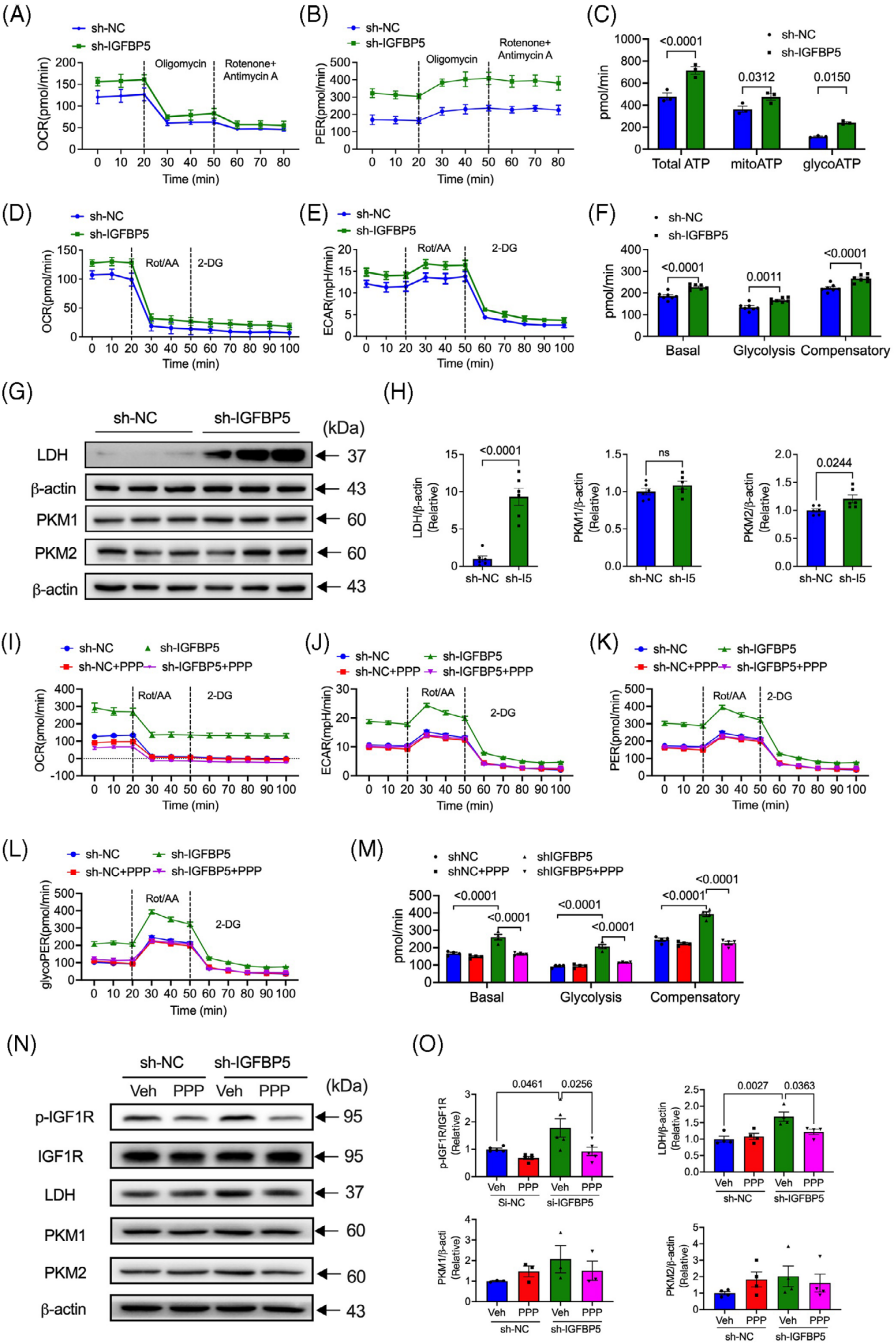
Insulin‐like growth factor‐binding protein 5 (IGFBP5) deficiency promotes glycolysis through IGF1R. (A) OCR and (B) ECAR profiles in sh‐NC‐ and sh‐IGFBP5‐infected human umbilical vein endothelial cells (HUVECs) (*n* = 8 in each group). (C) Quantification of ATP production in sh‐NC‐ and sh‐IGFBP5‐infected HUVECs. (D) OCR and (E) ECAR profiles showing the glycolytic function in sh‐NC‐ and sh‐IGFBP5‐infected HUVECs (*n* = 8 in each group). (F) Quantification of glycolytic function parameters in sh‐NC‐ and sh‐IGFBP5‐infected HUVECs (*n* = 8 in each group). (G) Western blotting images and (H) quantification of the expression of lactate dehydrogenase (LDH), PKM1 and PKM2 in sh‐NC‐ and sh‐IGFBP5‐infected HUVECs. (I) OCR, (J) ECAR, (K) proton efflux rate (PER) and (L) glycoPER profiles showing glycolytic function in sh‐NC‐ and sh‐IGFBP5‐infected HUVECs in the presence or absence of IGF1R inhibitor picropodophyllin (PPP) (*n* = 6 in each group). (M) Quantification of glycolytic function parameters in sh‐NC‐ and sh‐IGFBP5‐infected HUVECs in the presence or absence of PPP (*n* = 8 in each group). (N) Western blot images and (O) quantification of the expression of LDH, PKM1 and PKM2 in sh‐NC‐ and sh‐IGFBP5‐infected HUVECs in the presence or absence of IGF1R inhibitor PPP (*n* = 6 in each group).

Endothelial cell metabolism is a critical driving force of angiogenesis.^39^ Therefore, we assessed mitochondrial consumption by testing the oxigen consumption rate (OCR) and extracellular acidification rate (ECAR) in IGFBP5‐shRNA‐infected HUVECs using a Seahorse Extracellular Analyser. As shown in the OCR and ECAR curves for ATP production, IGFBP5‐shRNA infection induced an appreciable increase in the OCR and ECAR (Figure 6A,B). Meanwhile, the total ATP production and mitochondrial ATP were increased in IGFBP5‐shRNA‐infected cells compared to the control cells (Figure 6C). Most importantly, glycolytic ATP in IGFBP5‐shRNA‐infected cells was significantly elevated compared to the energy generation via mitochondria. Proliferating cells dominantly generate energy by generating a high rate of glycolysis and lactic acid fermentation in the cytosol. Thus, we further investigated glycolysis and found a significant elevation in the basal, glycolysis and compensatory glycolysis in IGFBP5‐shRNA‐infected cells (Figure 6D‒F). Glycolysis‐associated enzymes, including pyruvate kinase muscle isozyme (PKM) and LDH, are critical regulators of glycolysis production.^40^, ^41^, ^42^, ^43^, ^44^ Therefore, to elucidate the pathway that participated in glycolysis production modulated by IGFBP5, we measured the expression levels of LDH, PKM1 and PKM2. LDH expression significantly increased in IGFBP5‐KD cells. In contrast, we observed no changes in the expression levels of PKM1 or PKM2 (Figure 6G,H).

Our previous results showed that IGF1R is a critical pathway in IGFBP5‐regulated cell functions, including cell proliferation, tube‐like formation and cell migration. We then determined the role of IGF1R in glycolytic metabolism using an IGF1R‐specific antagonist, PPP (AXK1717). Interestingly, the enhanced glycolytic ATP production in IGFBP5‐shRNA‐infected cells was decreased by PPP (Figure 6I‒M). Moreover, shIGFBP5‐promoted phosphorylation of the IGF1R was inhibited by PPP and LDH expression (Figure 6N,O). In addition, we also applied glycolysis inhibitors, specific blockers of PFKFB3, 3‐(3‐pyridinyl)−1‐(4‐pyridinyl)−2‐propen‐1‐one (3‐PO) and a non‐metabolizable glucose analogue 2‐DG, to validate the connection between IGFBP5 and the angiogenic effect through glycolysis. The results showed that IGFBP5 deficient‐induced angiogenesis marketable decreased by treating with 3‐PO and 2‐DG (Figure S4). These results suggested that IGFBP5 mediated glycolytic metabolism and that suppression of IGF1R with PPP reverses IGFBP5 deficiency‐induced metabolism.

### 3.7 IGFBP5 exerts its role via E3 ubiquitin ligase VHL‐regulated HIF1α stability

**FIGURE 7:**
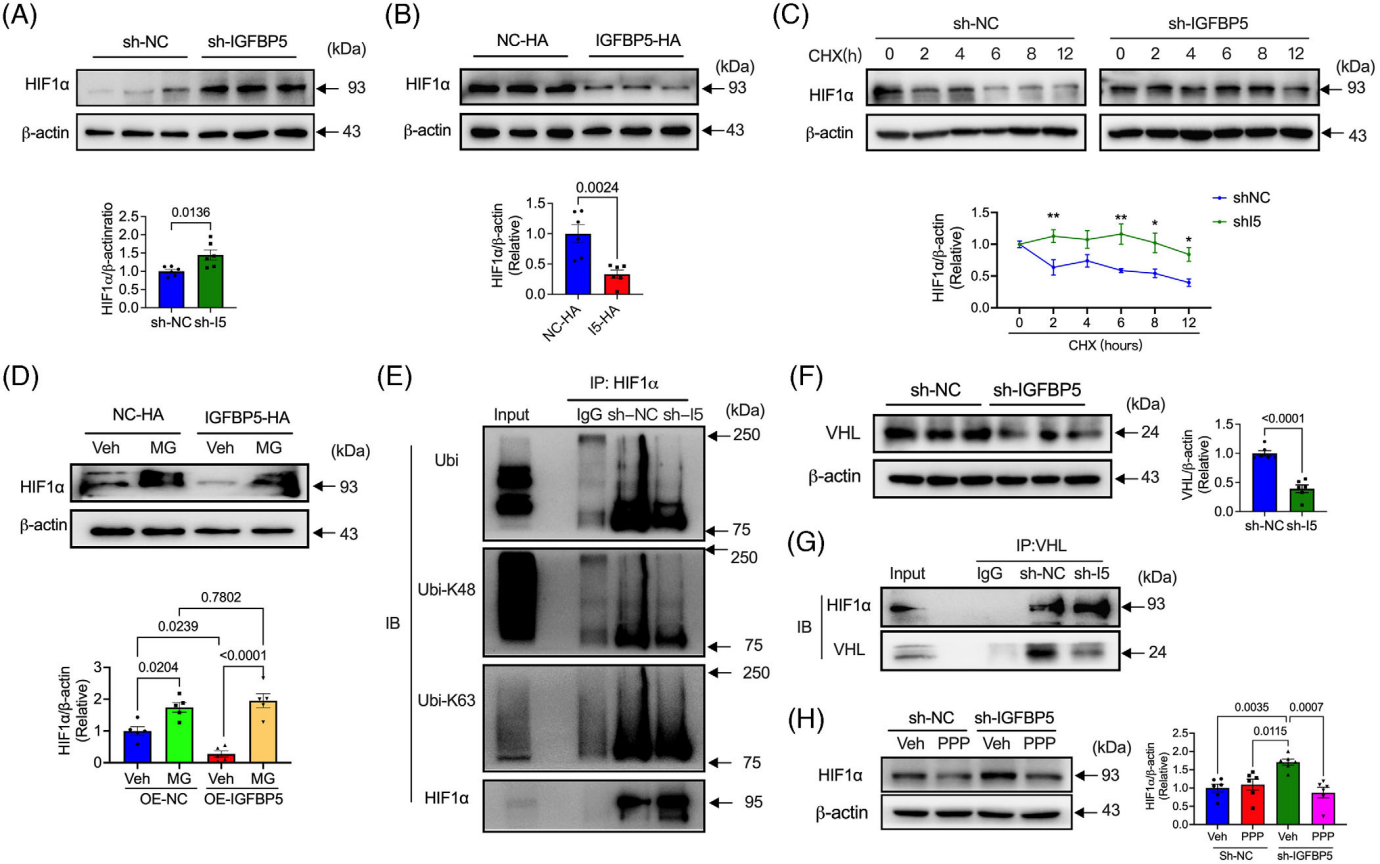
Deletion of insulin‐like growth factor‐binding protein 5 (IGFBP5) increases HIF1α expression by suppressing ubiquitination via ubiquitin ligase Von Hippel‐Lindau (VHL). (A) Western blotting images and quantification of HIF1α in sh‐NC‐ and sh‐IGFBP5‐infected human umbilical vein endothelial cells (HUVECs) (*n* = 6 in each group). (B) Western blotting images and quantification of HIF1α in NC‐overexpression (OE‐NC)‐ and IGFBP5‐overexpression (OE‐IGFBP5)‐transfected HUVECs (*n* = 6 in each group). (C) The stability of HIF1α expression in sh‐NC‐ and sh‐IGFBP5‐transfected HUVECs in the presence of 20 μg/mL cycloheximide (CHX) determined by Western blot (*n* = 5 in each group). (D) The stability of HIF1α expression in OE‐NC‐ and OE‐IGFBP5‐transfected HUVECs in the presence of MG132 (MG) determined by Western blot. (E) Western blotting images of total ubiquitin, K48 ubiquitin and K63 ubiquitin of HIF1α in sh‐NC‐ and sh‐IGFBP5‐infected HUVECs (*n* = 3). (F) Western blotting images and quantification of VHL in sh‐NC‐ and sh‐IGFBP5‐infected HUVECs (*n* = 5 in each group). (G) Co‐immunoprecipitation of the interaction between VHL and HIF1α detected by Western blot in sh‐NC‐ and sh‐IGFBP5‐infected HUVECs (*n* = 3). (H) Western blot images and quantification of HIF1α expression in sh‐NC‐ and sh‐IGFBP5‐infected HUVECs in the presence or absence of IGF1R inhibitor picropodophyllin (PPP) (*n* = 5 in each group).

HIF1α is a key transcription factor that modulates angiogenesis and glycolysis by stimulating the glycolysis‐associated enzymes.^45^ To investigate the potential mechanism by which ablation of IGFBP5 affects angiogenesis and glycolytic metabolism, we probed whether IGFBP5 could regulate the HIF1α protein levels. Interestingly, the KD of IGFBP5 in HUVECs markedly promoted endogenous HIF1α (Figure 7A), whereas overexpression of IGFBP5 led to a reduction in cellular HIF1α expression (Figure 7B). Since HIF1α steady‐state levels are regulated by ubiquitin‐mediated proteasomal degradation,^46^ we used CHX, a protein synthesis inhibitor. After treatment with CHX, in control siRNA‐transfected HUVECs, the expression of HIF1α was reduced time‐dependent (Figure 7C). However, in sh‐IGFBP5‐transfected HUVECs, these decreases were suppressed significantly (Figure 7C), indicating that IGFBP5 can impact the HIF1α stability. To further investigate whether HIF1α stability is regulated by ubiquitin, we applied MG132, a blocker for ubiquitin‐proteasome degradation, in the IGFBP5‐overexpressed HUVECs. As shown in Figure 7D, the HIF1α level decreased significantly after overexpression of IGFBP5 and was promoted by inhibition of the ubiquitin‐proteasome by MG132. However, IGFBP5 overexpression did not affect the MG132‐promoted HIF1α expression (Figure 7D), implying ubiquitin‐proteasome participation in IGFPB5‐regulated HIF1α stability. Furthermore, we investigated the HIF1α ubiquitination in IGFBP5‐shRNA‐infected cells. As shown in Figure 7E, HIF1α ubiquitination was restrained after the IGFBP5‐KD and mainly suppressed K48‐ and K63‐linked polyubiquitination. Additionally, a previous study has proved that the ubiquitin‐mediated proteasomal degradation of HIF1α employs the E3 ubiquitin ligase VHL.^46^ Therefore, when probed for VHL, our results showed that the VHL expression was suppressed by sh‐IGFBP5, indicating that VHL participated in IGFBP5‐mediated HIF1α ubiquitination (Figure 7F). Moreover, the IGFBP5‐regulated interaction between HIF1α and VHL was also observed (Figure 7G). Since our previous results have proved that IGF1R is the critical regulator in mediating the role of IGFBP5, further investigations demonstrated that IGFBP5‐promoted HIF1α expression was suppressed by the IGF1R inhibitor PPP (Figure 7H).

### 3.8 Inhibition of IGF1R abolished the angiogenesis‐associated function of IGFBP5‐EKO

To validate whether the in vitro molecular pathway is also involved in IGFBP5^EKO^ mice under the HLI injury, we also tested the Ki67 (cell proliferation marker), Akt, Erk1/2, IGF1 and HIF1α expressions in the mice. The expression of Ki67 (Figure S5A,B), pAkt (Figure S6A,B), pErk1/2 (Figure S6C,D), HIF1α (Figure S7A,B) and IGF1R (Figure S7C,D) were all increased in IGFBP5^EKO^ mice compared to IGFBP5^f/f^ mice post‐HLI.

**FIGURE 8:**
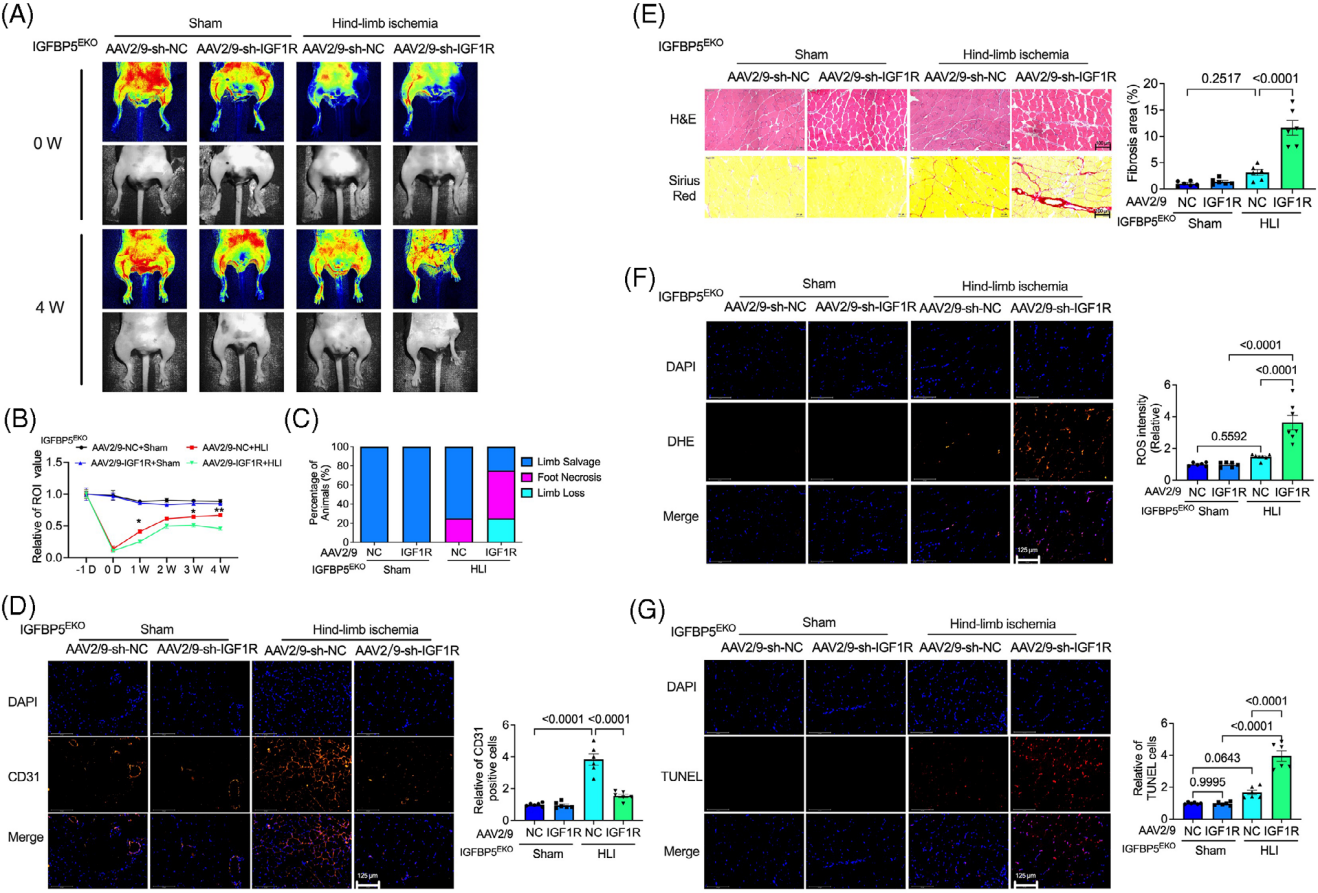
Endothelial cell (EC)‐specific deletion of IGF1R abolished IGFBP5^EKO^‐attenuated limb ischaemia. (A) Representative images detected by laser Doppler‐based tissue perfusion system in IGFBP5^EKO^ mice, which were infected with AAV2/9‐sh‐NC or AAV‐sh‐IGF1R after ischaemia induction (0 W) and 4 weeks after ischaemia (4 W). (B) Quantification of blood flow in the hindlimb of IGFBP5^EKO^ mice infected with AAV2/9‐sh‐NC or AAV‐sh‐IGF1R before (−1 D) and after ischaemia induction at 0 days, and 1, 2, 3 and 4 weeks (*n* = 8 in each group). (C) Percentage of limb salvage, foot necrosis and limb loss in sham or ischaemic IGFBP5^EKO^ mice infected with AAV2/9‐sh‐NC or AAV2/9‐sh‐IGF1R 4 weeks after ischaemia induction (*n* = 8 in each group). (D) Representative images of immunofluorescence staining, and quantification of CD31 (red) and DAPI (blue) in sham or ischaemic IGFBP5^EKO^ mice infected with AAV2/9‐sh‐NC or AAV2/9‐sh‐IGF1R 4 weeks after ischaemia induction (*n* = 5 in each group). (E) Representative images of haematoxylin‒eosin and picrosirius red staining, and quantification of fibrosis area in gastrocnemius of the hindlimb in ischaemic or sham in IGFBP5^EKO^ mice infected with AAV2/9‐sh‐NC or AAV2/9‐sh‐IGF1R 4 weeks after ischaemia induction (*n* = 5 in each group). (F) Representative immunofluorescence staining images and quantification of dihydroethidium (DHE) staining for reactive oxygen species (ROS) detection in gastrocnemius of the hindlimb in sham or ischaemic IGFBP5^EKO^ mice infected with AAV2/9‐sh‐NC or AAV2/9‐sh‐IGF1R 4 weeks after ischaemia induction (*n* = 5 in each group). (G) Representative immunofluorescence staining images and quantification of TUNEL staining in gastrocnemius of the hindlimb in sham or ischaemic IGFBP5^EKO^ mice infected with AAV2/9‐sh‐NC or AAV2/9‐sh‐IGF1R 4 weeks after ischaemia induction (*n* = 5 in each group).

To examine the crucial effect of the IGF1R in IGFBP5‐regulated angiogenesis and hindlimb repair, we constructed an EC‐specific IGF1R‐deficiency adeno‐associated virus (AAV2/9‐Tie‐IGF1R‐shRNA) and infected it with sham or ischaemic IGFBP5^EKO^ mice. The expression of IGF1R was remarkably decreased in the ischaemic tissue after being infected with AAV2/9‐IGF1 shRNA (Figure S8A,B). Interestingly, the IGFBP5^EKO^‐accelerated restoration of blood flow and repair of the ischaemia was partly suppressed by IGF1R‐shRNA (Figure 8A‒C). Moreover, IGF1R‐shRNA also reduced the IGFBP5^EKO^‐promoted neovascularisation (Figure 8D). Additionally, the protective effects observed in IGFBP5^EKO^, including decreased inflammation and fibrosis (Figure 8E) in the perivascular region of the gastrocnemius, reduced ROS production (Figure 8F) and apoptotic cells (Figure 8G) in the gastrocnemius, were neutralised by IGF1R‐shRNA. These data imply an essential role of the IGF1R in IGFBP5‐regulated angiogenesis and ischaemic hindlimb repair.

**FIGURE 9:**
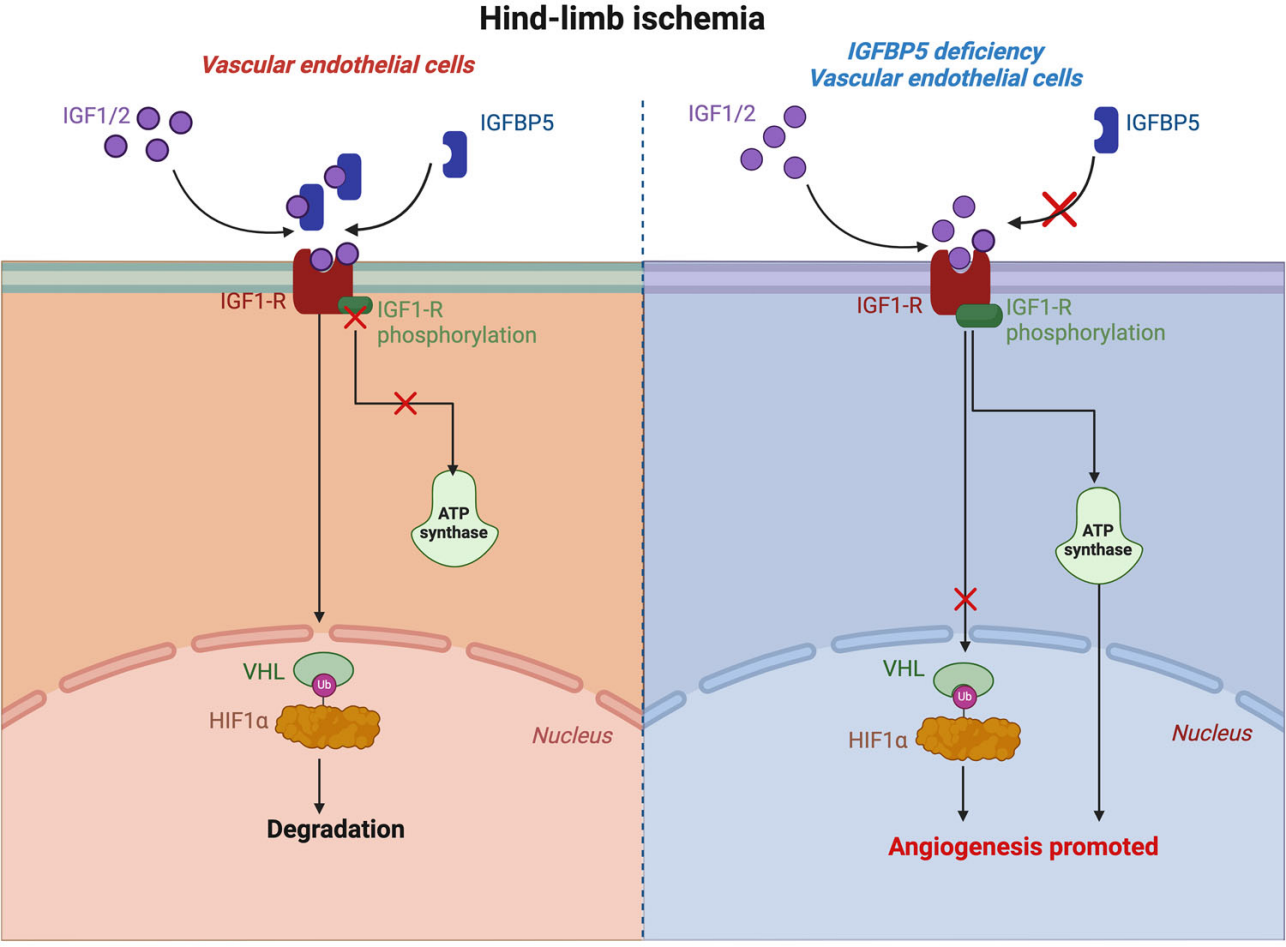
Schematic illustration describing the underlying mechanism of insulin‐like growth factor‐binding protein 5 (IGFBP5)‐regulated endothelial cell angiogenesis post‐hindlimb ischaemia (HLI). After HLI, IGFBP5 production was promoted, which inhibited the action of IGF1/2 and the phosphorylation of IGF1R and ATP production, thereby promoting the ubiquitin‐ligase Von Hippel‐Lindau (VHL) and the ubiquitination and degradation of HIF1α. When deficiency of IGFBP5 in endothelial cells (ECs), the IGF1R phosphorylation was promoted, the ATP production was promoted, and the ubiquitination of HIF1α was suppressed, which promoted angiogenesis post‐HLI.

## 4 DISCUSSION

In the present study, we explored the characterisation of IGFBP5 in the pathology of angiogenesis and attempted to dissect the underlying molecular mechanisms. As shown in Figure 9, the key findings of the study are: (1) IGFBP5 is induced during reparative angiogenesis in a limb ischaemia model and the ischaemic area of patients with CLI, as well as in hypoxic ECs. (2) In vivo deletion of IGFBP5 in the endothelium inhibits the deterioration of ischaemia‐induced limb salvage and blood perfusion. (3) IGFBP5 negatively regulates tube formation, cell proliferation and migration by blocking IGF signalling via the IGF1R/Akt/Erk1/2 axis in HUVECs by mediating glycolytic metabolism via IGF1R. (4) Mechanistically, IGFBP5 exerts these functions by regulating the HIF1α stability via E3 ubiquitin ligase VHL. (5) KD of IGF1R in ECs abolished the effect of IGFBP5 deficiency on ischaemic hindlimb repair.

IGFs and IGFBPs are increasingly recognised as essential regulators of cellular processes, including cell development, proliferation and migration.^47^, ^48^, ^49^, ^50^, ^51^, ^52^ As the most conserved member of the IGFBP family, IGFBP5 is broadly distributed in embryonic and adult tissues. Additionally, the level of its expression is high in a variety of disorder‐related tissues, such as in severe chronic obstructive pulmonary disease,^53^ amniotic fluid of pregnant women with congenital heart disease,^54^ G2 tumour‐grade breast cancer,^55^ breast cancer in populations of African descent,^56^ Alzheimer's disease patients^57^ and glioblastoma multiforme.^58^ However, the role of IGFBP5 in cardiovascular physiology and pathology has rarely been investigated. Here, we found that the expression of IGFBP5 was markedly promoted in the perivascular gastrocnemius both in the ischaemic hindlimb of mice and CLI of patients, indicating that the upregulation of IGFBP5 in the vasculature is significantly related to the pathological ischaemic limb.

In addition, IGFBP5 is critically associated with the proliferation and motility of cancer cells and others, such as breast cancer cells,^59^ glioblastoma,^58^ nucleus pulposus cells,^60^ dental pulp stem cells,^61^ periodontal ligament stem cells and Wharton's jelly of umbilical cord stem cells.^62^ Increasing evidence has shown that regulating abnormally expressed IGFBP5 may contribute significantly to these disorders. Hence, to further investigate the function of IGFBP5 in ECs, we produced EC‐specific IGFBP5‐KO mice. In IGFBP5‐EKO mice, IGFBP5 expression was substantially decreased in ECs. Moreover, IGFBP5‐EKO mitigated limb loss or necrosis induced by ischaemia, increased the blood flow in the ischaemic area and inhibited apoptosis and fibrosis in the perivascular gastrocnemius. IGFBP5‐EKO increased CD31 expression, which may be not only a function of neovascularisation but also a result of protection from injury by protection from ROS and apoptosis. Furthermore, in vitro investigations revealed that the KD of IGFBP5 promoted tube‐like formation, cell proliferation and migration in HUVECs by activating the IGF1/Akt/Erk1/2 pathways. In contrast, the administration of rhIGFBP5 in HUVECs resulted in an opposing effect by decreasing tube formation, cell proliferation and migration.

There is considerable evidence of the essential role of IGFs and their signal transduction networks in regulating energy metabolism and growth during oncogenesis.^63^ The IGF family is comprised of three ligands (INS, IGF1 and IGF2), three cell surface receptors (INSR, IGF1R and IGF2R) and 10 IGF‐binding proteins (IGFBP1‒IGFBP7 and IGF2BP1‒IGF2BP3).^52^ As a member of the IGFBPs family, IGFBP5 binds with high affinity to circulate IGFs.^64^ Additionally, IGFBP5 blocks IGF signalling by binding to IGF and preventing its interaction with IGF1R^35^; once secreted, IGFBP5 works alongside other intracellular feedback mechanisms to inhibit the role of IGF1 and serves as a non‐cell autonomous feedback mechanism for tumours to reduce IGF1R signalling in the adjacent normal cells.^51^ However, whether IGFBP5 positively or negatively affects IGF1 signalling remains debatable. One possibility is that IGFBP5 may enhance the function of IGF1, presumably by efficiently presenting IGF1 to IGF1R.^65^ Transgenic expression of a mutant IGFBP5 encoding a variant having reduced binding affinity for IGF did not lead to skeletal abnormalities, indicating that the adverse effects observed on skeletal development are associated with a mechanism dependent on IGF binding.^66^ Consistent with these observations, our findings confirmed that IGFBP5 binds to and blocks the IGF1 and IGF2 function in regulating tube formation, cell proliferation and migration via the IGF1R/Akt/Erk1/2 pathways.

A previous report demonstrated that IGF1R showed more levels and activation in non‐muscle‐invasive bladder tumours than in muscle‐invasive ones. Moreover, IGF1R phosphorylation in tumours shows a significant inverse correlation with IGFBP5.^67^ Consistent with this finding, we also found that in HUVECs, rhIGFBP5 restrained the IGF1R phosphorylation, whereas IGFBP5‐KD promoted it. In addition to tumours, elevated IGFBP5 levels were detected in diabetic kidney disease (DKD) mice. Also, knocking out IGFBP5 reduced kidney inflammation in DKD mice by modulating glycolysis.^17^ To understand the roles of IGFBP5 further, our research has found that the KD of IGFBP5 in ECs promoted ATP production during glycolysis, which can be reduced by a specific IGF1R blocker (PPP) via regulating LDH and HIF1α.

HIF1α is critical in mediating the cellular responses towards hypoxia. These responses include regulating genes associated with energy metabolism, angiogenesis and apoptosis. To date, this is the most widely investigated angiogenesis signalling.^68^ Under normal conditions, HIF1α expression gets rapidly degraded by the proteasomes, whereas it remains stable under hypoxia.^46^ However, under normoxia, the prolyl hydroxylases hydroxylate two conserved proline residues on HIF1α, which results in the binding of VHL, the recognition element of a ubiquitin‒ligase complex, thereby, inducing HIF1α ubiquitylation and degradation.^69^ Since IGFBP5 is reported as a direct transcriptional target of HIF1α, it may serve as a feedback mechanism for tumours to inhibit IGF1R signalling in adjacent normal cells.^51^ In the present investigation, we discovered that HUVECs with an IGFBP5 deficiency had significantly higher expression of HIF1α. The molecular mechanism underlying this process may be that IGFBP5 regulates the HIF1α stability that relies on E3 ubiquitin ligase VHL. Although our findings provide a novel VHL‐dependent mechanism for enhancing stability by deleting IGFBP5, the detailed molecular mechanisms underlying this process require further investigation.

## 5 CONCLUSIONS

In summary, as a result of its actions on IGF1R/HIF1α/Akt/Erk1/2 pathways, we demonstrated IGFBP5 as a crucial regulator in ischaemia‐induced angiogenesis. Since several studies performed so far have demonstrated the diverse effects of IGFBP5 in the regulation of cell function, our data implied that IGFBP5 might perform as an important therapeutic agent for disorders in humans, including diabetic nephropathy and CLI. However, there are a few weaknesses in this study. First, further study should be carried out to identify the angiogenesis effect of IGFBP5 on improving myocardial infarction. Second, the detailed molecular mechanism of IGFBP5‐regulated VHL‐dependent HIF1α activity needs to be clarified. Another challenge is that using gene editing technology to process IGFBP5 to treat clinical ischaemic diseases still requires a series of research.

## AUTHOR CONTRIBUTIONS

Gang Li and Yan Wang designed the experiments. Fei Song and Yu Hu performed most of the experiments. Gang Li wrote the manuscript. Gang Li and Yan Wang supervised the study and revised the manuscript. Gang Li, Yan Wang and Wei‐Yin Wu provided funding. Hu Sun collected the human CLI samples. Yu Hu, Yi‐Xiang Hong, Yue Han and Yi‐Jie Mao performed investigations associated with animal samples. All authors contributed to the writing and editing of the manuscript.

## CONFLICT OF INTEREST STATEMENT

The authors declare they have no relevant financial or non‐financial interests to disclose.

## DATA AVAILABILITY STATMENT

All data supporting the findings of this study are available in the article and Supporting Information. Additional data related to this paper may be requested from the authors.

## ETHICS STATEMENT

All animal studies were conducted following the Declaration of Helsinki and approved by the Animal Care Committee of Xiamen University (approval no. XMULAC20190120).

## Supporting information

Supporting Information
