# Supplementary material for: Deletion of endothelial IGFBP5 protects against ischaemic hindlimb injury by promoting angiogenesis
Source: Clin Transl Med. 2024 Jun 17;14(6):e1725. doi: 10.1002/ctm2.1725 (PMC11182737; doi:10.1002/ctm2.1725)
Supplement: Supplementary file 1 — Supporting Information [file CTM2-14-e1725-s001.docx]

**Supplemental Material**

**Deletion of Endothelial IGFBP5 Protects Against Ischemic Hindlimb Injury by Promoting Angiogenesis**

**Supplemental Table 1. Demographic details of patients**

| Patient number | Age | Weight | Gender | Disease |
| --- | --- | --- | --- | --- |
| 1 | 65 | 72 | 1 | arteriosclerosis obliterans of lower extremities |
| 2 | 56 | 42 | 2 | arteriosclerosis obliterans of lower extremities |
| 3 | 65 | 62 | 1 | arteriosclerosis obliterans of lower extremities |
| 4 | 50 | 60 | 1 | arteriosclerosis obliterans of lower extremities |
| 5 | 72 | 45.5 | 1 | iliac artery occlusion |
| 6 | 61 | 35 | 1 | arteriosclerosis obliterans of lower extremities |

**Supplemental Figure and Figure Legends**

**Figure S1. Identification of Endothelial-specific IGFBP5 knockout mice.**

**
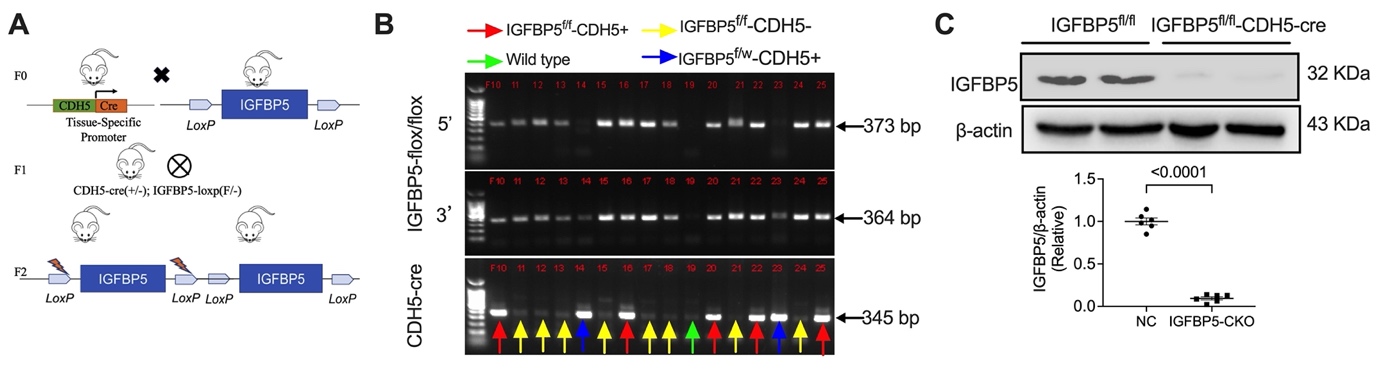
A,** Construct used to generate an endothelial-specific IGFBP5 mouse. **B,** genotyping of Endothelial-specific IGFBP5 knockout mice. Flox bands (Top, 5′-Flox^+/+^, 373 bp; Middle: 3′ Flox^+/+^, 364 bp) and Cdh5-Cre bands (Bottom, Cre^+^, 345 bp). Yellow arrows indicated IGFBP5^fl/fl^ -Cre-negative mice**,** red arrows indicated the IGFBP5^fl/fl^-Cre-positive mice, green arrows indicated wild-type mice, blue arrows indicated IGFBP5^fl/wt^ -Cre-positive mice. **C,** representative western blot images and quantification of IGFBP5 in murine lung endothelial cells (MLECs) of IGFBP5^fl/fl^ and IGFBP5^fl/fl^ -Cdh5-Cre mice. Data are presented as relative fold change to IGFBP5 ^fl/fl^ (n = 6 mice per group). Statistical analysis was performed using Student’s t-test for **C**.

**Figure S2. Body weight of the IGFBP5 EKO mice compared to the control mice (IGFBP5^f/f^).**

**
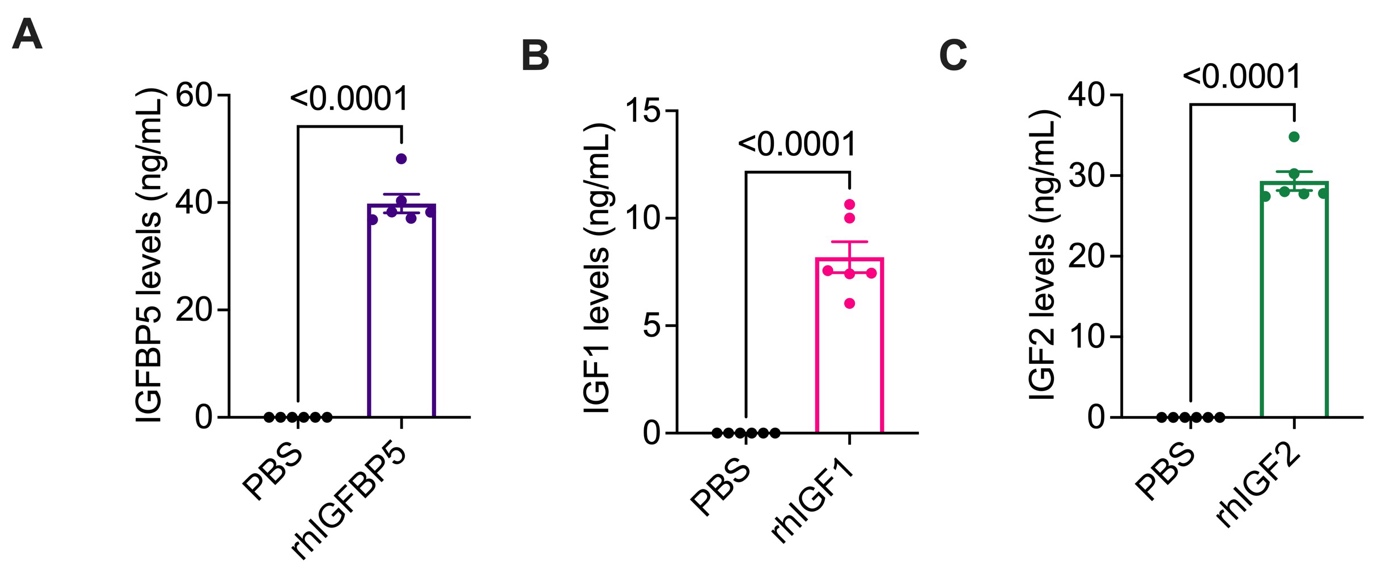
Figure S3. The concentration of IGFBP5, IGF1, and IGF2 in the medium after treatment with relative recombinant proteins. A,** ELISA assay detected IGFBP5 concentration after treatment with rhIGFBP5. **B,** ELISA assay detected IGF1 concentration after treatment with rhIGF1. **C,** ELISA assay detected IGF2 concentration after treatment with rhIGF2.

**
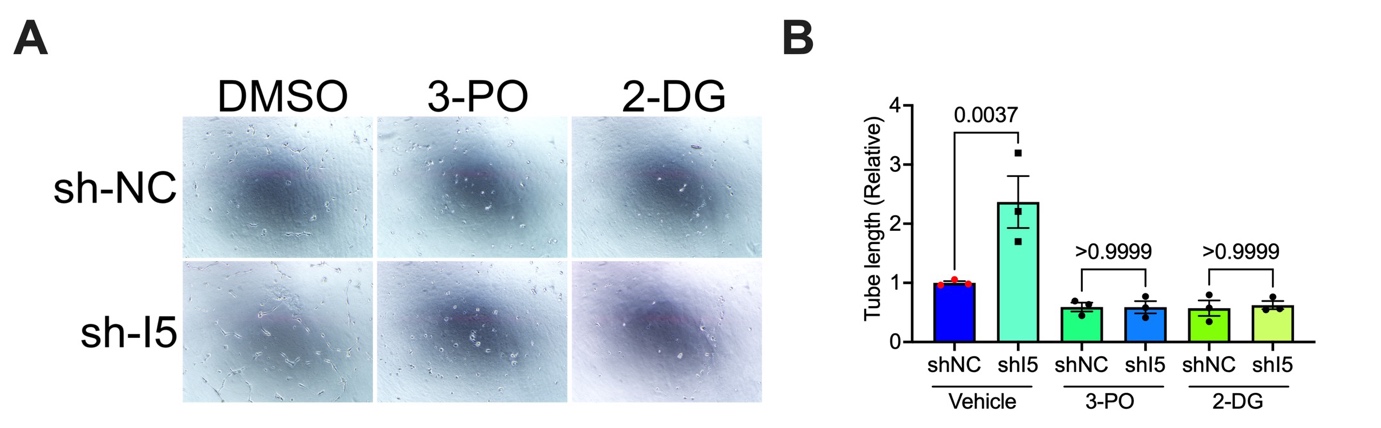
Figure S4. Tube formation after** **treatment with 3-PO and 2-DG** **in the sh-control- and sh-IGFBP5-infected HUVECs. A,** Representative images of tube formation in the control shRNA (sh-NC)- and IGFBP5 shRNA (sh-I5)-infected HUVECs treatment with 3-PO and 2-DG. **B,** Data analysis of tube length in the control shRNA (sh-NC)- and IGFBP5 shRNA (sh-I5)-infected HUVECs treatment with 3-PO and 2-DG.

**
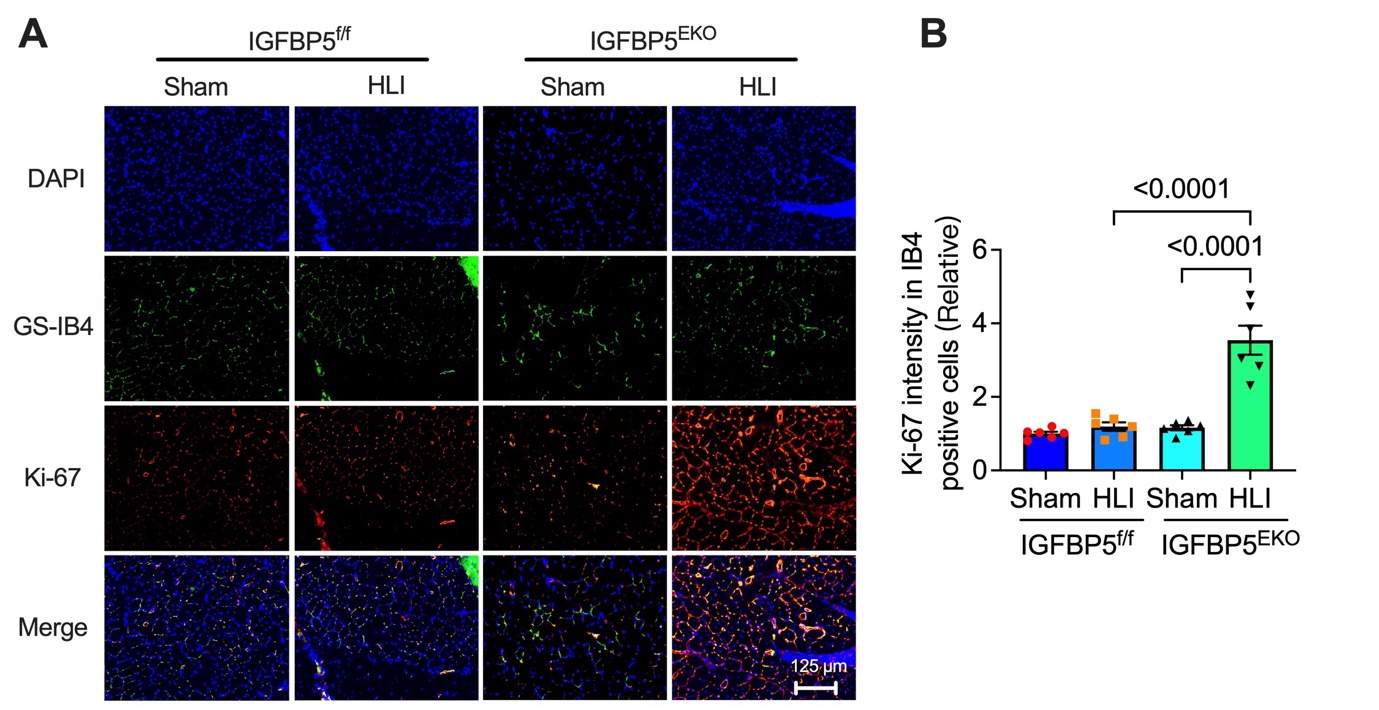
**

**Figure S5. The expression of proliferated endothelial cells in IGFBP5^EKO^ and IGFBP5^f/f^ mice performed HLI or sham surgery. A,** representative immunofluorescence staining images, and **B,** quantification of Ki-67 (red) in GS-IB4 positive cells (green) in gastrocnemius of hindlimb from IGFBP5^EKO^ and IGFBP5^f/f^ mice treated with sham or HLI (n = 6 in each group).

**
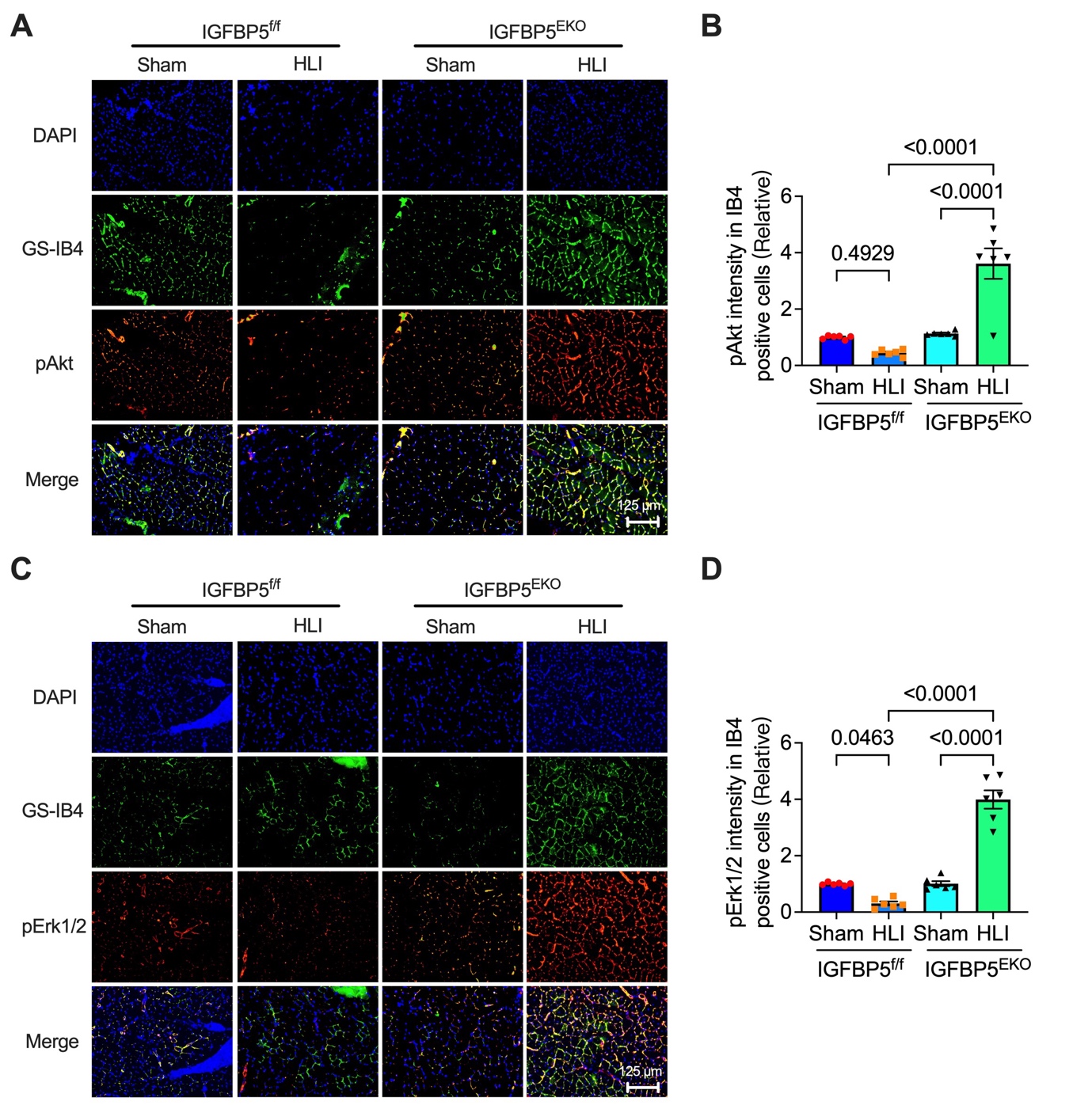
**

**Figure S6. The expression of Akt and Erk1/2 in endothelial cells of IGFBP5^EKO^ and IGFBP5^f/f^ mice performed HLI or sham surgery. A,** representative immunofluorescence staining images, and **B,** quantification of pAkt (red) in GS-IB4 positive cells (green) in gastrocnemius of hindlimb from IGFBP5^EKO^ and IGFBP5^f/f^ mice treated with sham or HLI (n = 6 in each group). **C,** representative immunofluorescence staining images, and **D,** quantification of pErk1/2 (red) in GS-IB4 positive cells (green) in gastrocnemius of hindlimb from IGFBP5^EKO^ and IGFBP5^f/f^ mice treated with sham or HLI (n = 6 in each group).

**
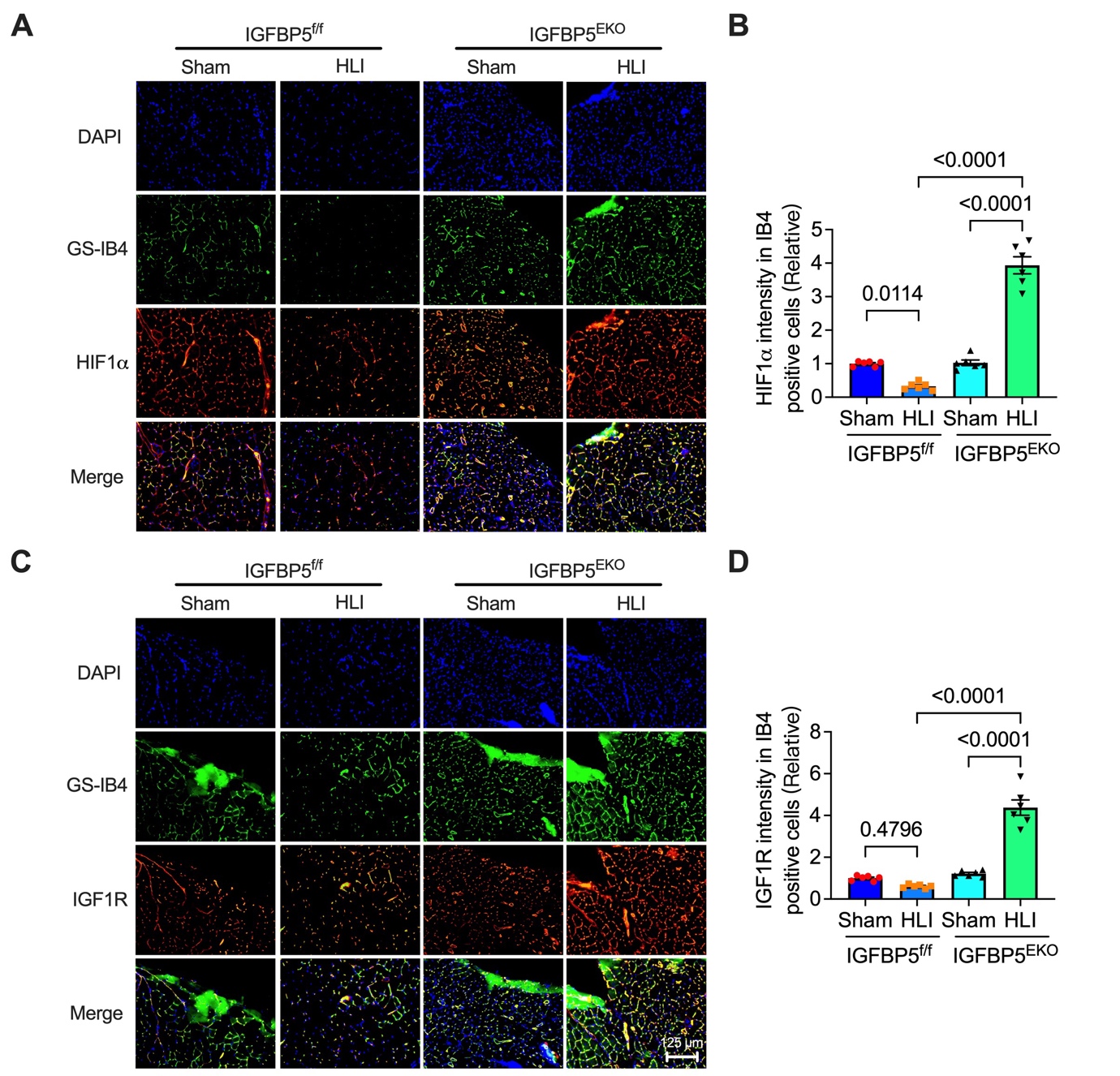
**

**Figure S7. The expression of** **HIF1α and IGF1R in endothelial cells of IGFBP5^EKO^ and IGFBP5^f/f^ mice performed HLI or sham surgery. A,** representative immunofluorescence staining images, and **B,** quantification of HIF1α (red) in GS-IB4 positive cells (green) in gastrocnemius of hindlimb from IGFBP5^EKO^ and IGFBP5^f/f^ mice treated with sham or HLI (n = 6 in each group). **C,** representative immunofluorescence staining images, and **D,** quantification of IGF1R (red) in GS-IB4 positive cells (green) in gastrocnemius of hindlimb from IGFBP5^EKO^ and IGFBP5^f/f^ mice treated with sham or HLI (n = 6 in each group).

**
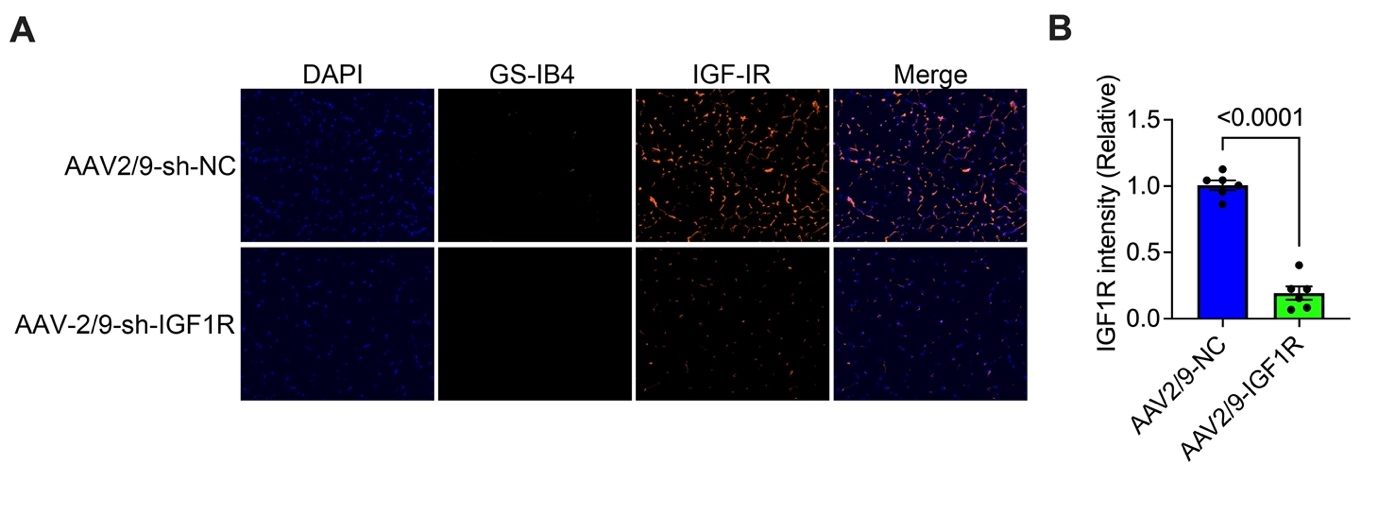
Figure S8. IGF1R expression** **in gastrocnemius muscle tissue of the mice infected with** **AAV2/9-control or AAV2/9-sh-IGF1R. A,** Immunofluorescence co-staining of GS-IB4 and IGF1R in gastrocnemius muscle tissues of AAV2/9-control- or AAV2/9-sh-IGF1R-infected mice. **B,** the relative intensity of the expression of IGF1R in AAV2/9-control- or AAV2/9-sh-IGF1R-infected mice.

**
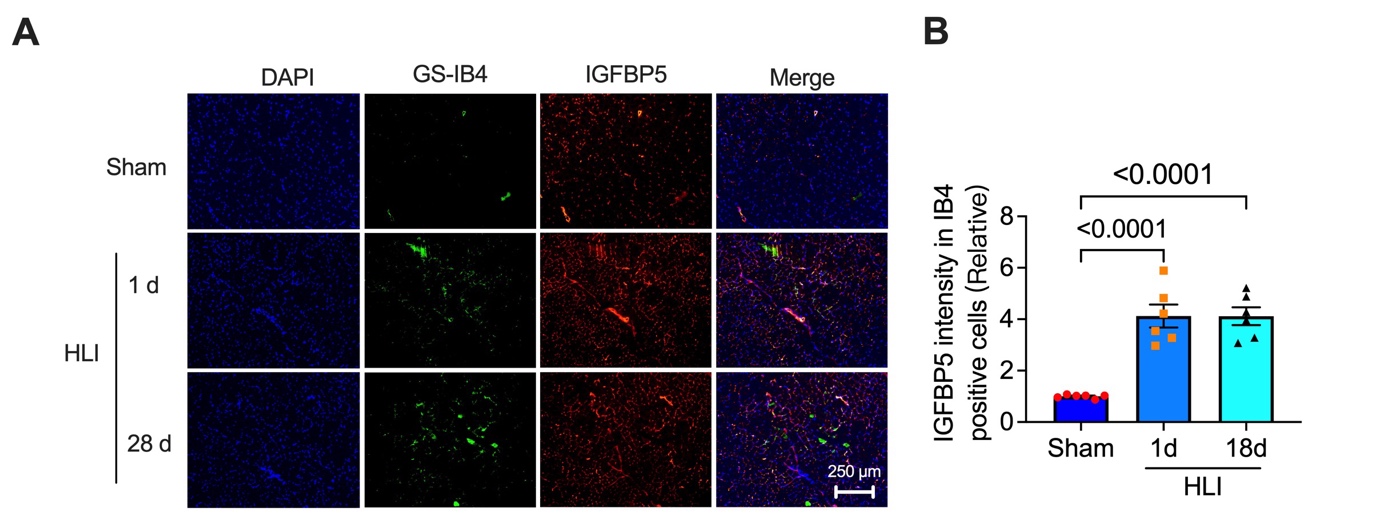
**

**Figure S9. IGFBP5 expression in gastrocnemius muscle tissue of the** **sham or HLI mice. A,** Immunofluorescence co-staining of GS-IB4 and IGFBP5 in gastrocnemius muscle tissues of sham or HLI mice. **B,** the relative intensity of the expression of IGFBP5 in the three groups.
